# Clathrin heavy chain 22 contributes to the control of neuropeptide degradation and secretion during neuronal development

**DOI:** 10.1038/s41598-018-19980-0

**Published:** 2018-02-05

**Authors:** Michael S. Nahorski, Georg H. H. Borner, Samiha S. Shaikh, Alexandra K. Davies, Lihadh Al-Gazali, Robin Antrobus, C. Geoffrey Woods

**Affiliations:** 1 Cambridge Institute for Medical Research, University of Cambridge, Cambridge, CB2 0XY UK; 2 Max Planck Institute of Biochemistry, Department of Proteomics and Signal Transduction, Am Klopferspitz 18, 82152 Martinsried, Germany; 3 Department of Peadiatrics, College of Medicine and Health Sciences, United Arab Emirates University, P.O.Box, 17666 Al-Ain, United Arab Emirates

## Abstract

The repertoire of cell types in the human nervous system arises through a highly orchestrated process, the complexity of which is still being discovered. Here, we present evidence that CHC22 has a non-redundant role in an early stage of neural precursor differentiation, providing a potential explanation of why CHC22 deficient patients are unable to feel touch or pain. We show the CHC22 effect on neural differentiation is independent of the more common clathrin heavy chain CHC17, and that CHC22-dependent differentiation is mediated through an autocrine/paracrine mechanism. Using quantitative proteomics, we define the composition of clathrin-coated vesicles in SH-SY5Y cells, and determine proteome changes induced by CHC22 depletion. In the absence of CHC22 a subset of dense core granule (DCG) neuropeptides accumulated, were processed into biologically active ‘mature’ forms, and secreted in sufficient quantity to trigger neural differentiation. When CHC22 is present, however, these DCG neuropeptides are directed to the lysosome and degraded, thus preventing differentiation. This suggests that the brief reduction seen in CHC22 expression in sensory neural precursors may license a step in neuron precursor neurodevelopment; and that this step is mediated through control of a novel neuropeptide processing pathway.

## Introduction

Clathrin is a molecular scaffold made up of self-assembling triskelions, recruited to membranes to sort proteins for intracellular trafficking^1^. There are two clathrin heavy chain proteins in humans, CHC22 and CHC17. The roles of the CHC17 clathrin heavy chain are wide ranging; it functions in numerous endosomal trafficking events, in mitotic spindle formation and during bacterial infection^2,3^. By contrast, the requirement for CHC22 has proven more puzzling. CHC17 and CHC22 have the same predicted structure and domains, and the human proteins display 85% identity; there are just a handful of potentially significant amino acid changes between the two proteins^2^. CHC17 is expressed at higher levels than CHC22 (between ten and one hundred fold) and yet CHC22 appears to have remained functional (indicated by the preservation of its protein length and functional domains) throughout most vertebrate evolution^4^. This suggests that CHC22 may have unique functions, not shared with CHC17.

CHC22 is expressed at its highest postnatal levels (but still much lower than CHC17) in muscle cells, where it mediates formation of the GLUT4 storage compartment^5^ by trafficking protein cargoes such as GLUT4 from endosomes to the trans-Golgi-network, independently of CHC17 or retromer^6^. However, the exact mechanism by which CHC22 controls this is unclear.

We recently identified a homozygous missense change in the gene that encodes CHC22 (*CLTCL1*) in children born unable to feel not only pain but also touch^7^. *CLTCL1* was significantly upregulated in the first trimester of the pre-natal developing human brain, and the p.E330K mutation present in the affected children caused a loss of function of the CHC22 protein in trafficking assays. CHC22 was unexpectedly found to be an essential negative regulator of sensory neuron differentiation, with a sudden fall in level during sensory precursor maturation preceding emergence of mature nociceptors^7^. However, the mechanism by which CHC22 acts during sensory neurodevelopment is unknown. Furthermore, this pre-natal CHC22 role appears distinct from a post-natal role of CHC22 in GLUT4 trafficking in muscle^5,6^.

The development of the peripheral (and central) nervous system is a complex and precisely orchestrated process involving spatial, temporal, autonomous and extraneous cues. Disruptions to this process can lead to congenital analgesia syndromes, in which patients have a complete lack of pain sensing from birth. A key shared feature of these syndromes is altered development or postnatal function of nociceptors, the primary pain sensing neurons. The two best understood syndromes are Hereditary Sensory and Autonomic type 4 (HSAN-IV) caused by *NTRK1* mutations^8^ and HSAN-V caused by *NGFB* mutations^9,10^. In both, painlessness results from disruption of the neurotrophic factor signalling induced by NGFβ binding to and activating the cell surface receptor TRKA^11^, which drives the differentiation and growth of nociceptors^12^. Hence, we hypothesised that CHC22 might also affect neurotrophic signalling during development of sensory neurons.

To investigate this, we have undertaken unbiased quantitative proteomics allied with trafficking and developmental cell biology studies. We find that CHC22 has a unique effect on paracrine/autocrine secretion sufficient to cause neuronal differentiation. Further, it regulates a hitherto unreported lysosomal trafficking pathway acting on a subset of neuropeptides transported in dense core granules. Downregulation of this pathway leads to increased secretion of neuropeptide cargo, allowing temporal control of SH-SY5Y differentiation. We hypothesise that disruption of this pathway by *CLTCL1* mutation could result in the loss of sensory neurons through early and ectopic precursor differentiation, leading to failure of neuron generation, and hence the deficiency of pain and touch sensing observed in patients.

## Results

### CHC22-mediated neural differentiation is independent of CHC17

**Figure 1:**
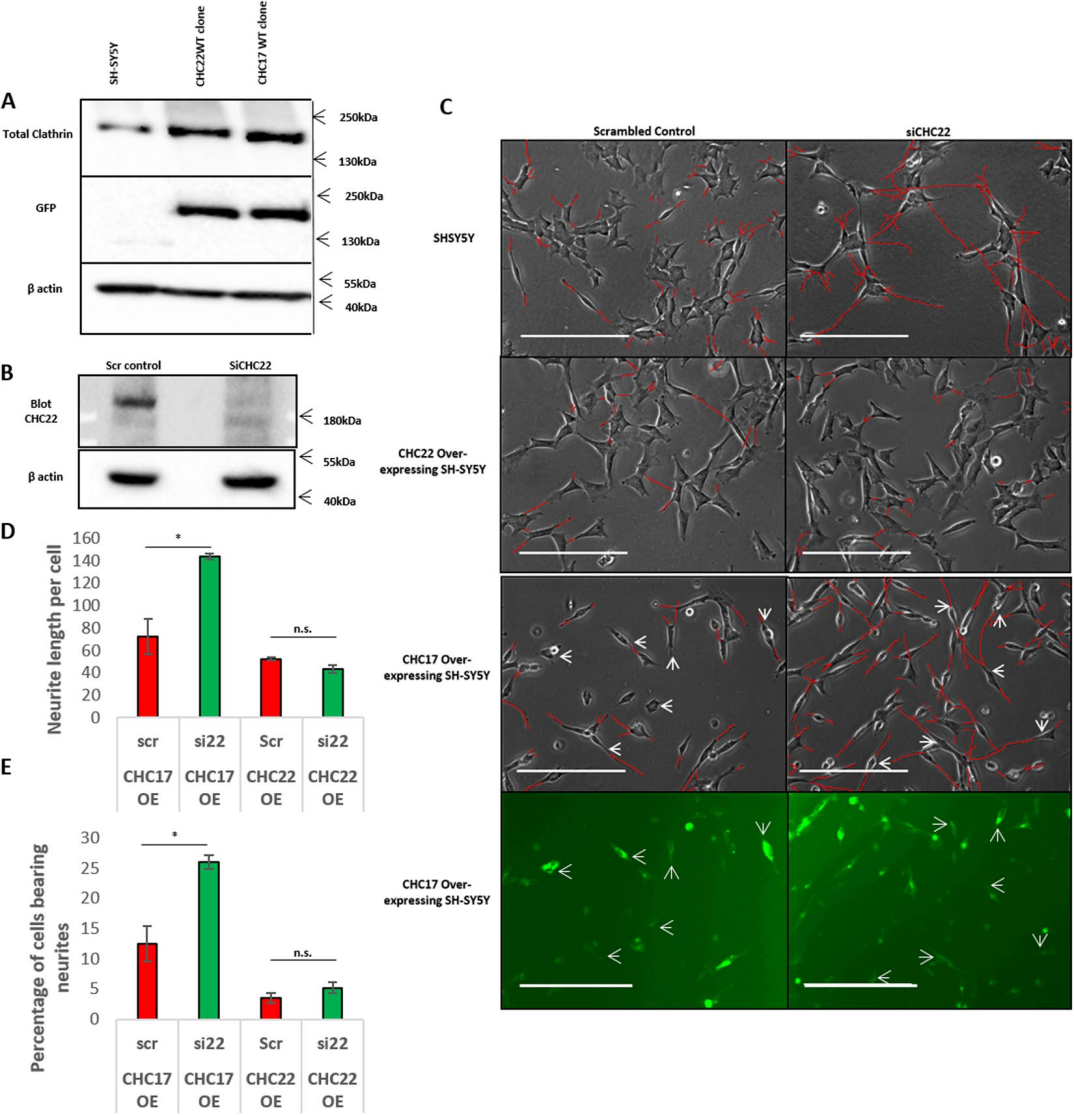
CHC17 is unable to rescue CHC22 knock-down induction of differentiation. (**A**) Expression of CHC22.GFP and CHC17.GFP in SH-SY5Y overexpressing cell lines. Comparison of total clathrin levels tested using the x22 antibody (**B**) Confirmation of CHC22 knockdown in CHC17 overexpressing cell line. (**C**) Phase contrast images of SH-SY5Y cells and clathrin over-expressing cell lines treated with 5 days (2 hits) CHC22 knockdown. White arrows highlight CHC17 overexpressing cells, identified by presence of GFP signal. Note presence of neurites (defined as >2 × cell body length) in parental cells and CHC17 overexpressing cells after CHC22 knockdown but not in those overexpressing CHC22.GFP. Scale bars represent 200 µm. Neurites have been traced in red to improve visibility. (**D**) Quantification of Neurite length per cell in CHC17 over expressing cells and CHC22 overexpressing cells. (**E**) Quantification of percentage of cells bearing neurites in CHC17 overexpressing cells and CHC22 overexpressing cells. Quantification of the Neurite length per cell and percentage of cells bearing neurites (greater than the length of two cell bodies) respectively according to methodology described in Nahorski *et al*.^7^. Significance tested by T test (Arcsine transformed for percentage data), one star represents (p < 0.05). Experiments carried out at least three independent times, error bars represent standard error, n.s. stands for not significant.

We have previously found that knockdown of CHC22 is sufficient to induce neurite outgrowth in SH-SY5Y cells; a neural-crest-derived cell line that can be differentiated with Retinoic acid and BDNF to resemble mature neurons *in vitr*o that express TRKA and TRKB (expressed in nociceptive and touch neurons respectively). Differentiation was also confirmed by an increase in βIII-Tubulin levels, see Fig. S9. We wanted to test whether this phenotype could be rescued by either CHC17 or CHC22, as CHC22 and CHC17 can be functionally redundant when expressed at artificially high levels, have the same predicted structure and domains, and the human proteins display 85% identity^13^. We therefore created a cell line stably overexpressing CHC22 and one stably overexpressing CHC17 (Fig. 1A) and subjected them to 5 day knockdown of CHC22. This confirmed that CHC22 overexpression restored the block to neurite induction, whereas overexpression of CHC17 did not (Fig. 1C–E). Thus, the effect of CHC22 as a negative regulator of neurite outgrowth is specific to CHC22 and not a result of flooding the cells with clathrin heavy chains *per se*.

### Depletion of CHC22 generates an autocrine/paracrine differentiation signal

**Figure 2:**
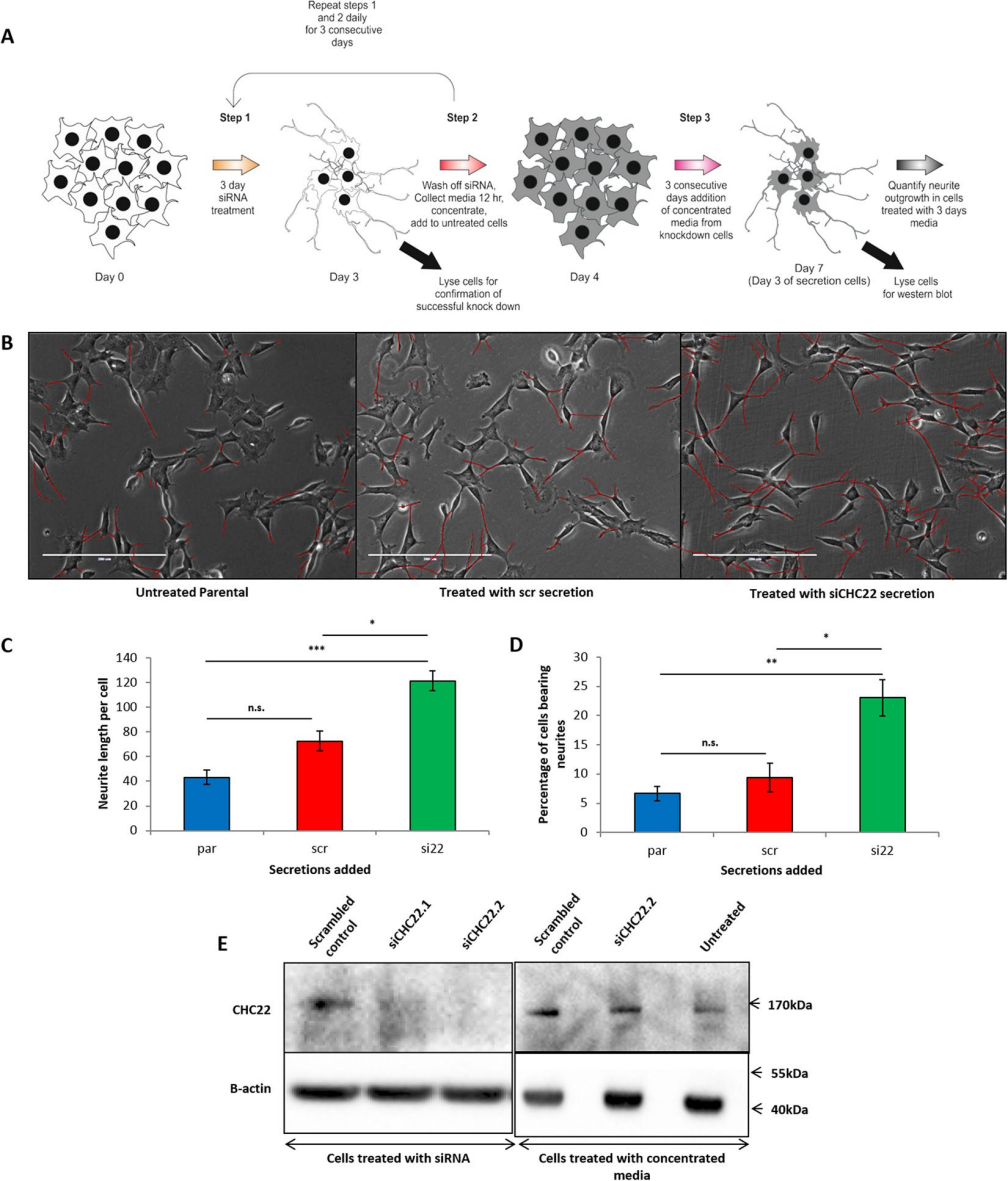
SH-SY5Y differentiation is driven by an autocrine secretory signal in CHC22 knockdown cells. (**A**) Schematic of experimental procedure collecting the media from cells with reduced CHC22 expression and adding it to fresh cells sequentially over 3 days. (**B**) Phase contrast images of cells spiked with the concentrated, secreted proteins from CHC22 knocked down cells and controls. Neurites quantified have been coloured in red to improve visualisation of the effect. (**C**) and (**D**) Quantification of the Neurite length per cell and percentage of cells bearing neurites (greater than the length of two cell bodies) respectively according to methodology described in Nahorski *et al*.^7^. Results represent at least three repeats. Statistics represent one way ANOVA (P < 0.001) with Bonferroni’s multiple comparison post hoc test (on Arcsine transformed data for percentages) (p < 0.05, p < 0.01, p < 0.001 are *, **, *** Respectively). (**E**) Western blot demonstrating the knockdown in cells from which secretions were collected and the CHC22 levels in cells differentiating in response to addition of these secretions.

We next asked if the neuronal differentiation seen after CHC22 depletion was caused by autocrine secretion or was cell autonomous^7^. We collected the secreted media from control or knockdown CHC22 cells each day for three consecutive days. We then added each daily secretion sequentially to control cells that had not been subject to knockdown (Fig. 2A). After three days, cells treated with the secretions from knockdown CHC22 cells differentiated; this was significant compared to untreated cells (3.4 fold increase in percentage of neurite bearing cells and 2.8 fold increase in neurite length per cell) (Fig. 2B–D). The differentiation observed after three days was as complete as that observed after 5 days of CHC22 siRNA knockdown.

### Quantitative proteomics demonstrates CHC22 knockdown increases levels of DCG proteins

We next sought to determine the components of the differentiation signal, and asked if CHC22 levels alter vesicle cargo transport of clathrin-coated vesicles during neuronal differentiation. We employed “Vesicle Fractionation Profiling” methodology^14^ to give an unbiased view of the proteins residing within clathrin coated vesicles (CCVs) in neuronal cells. This method combines quantitative proteomics with subcellular fractionation and uses cluster analysis to provide signatures of protein abundance in specific subcellular compartments. It also has been recently performed on a rodent neuronal cell line^15^.

**Figure 3:**
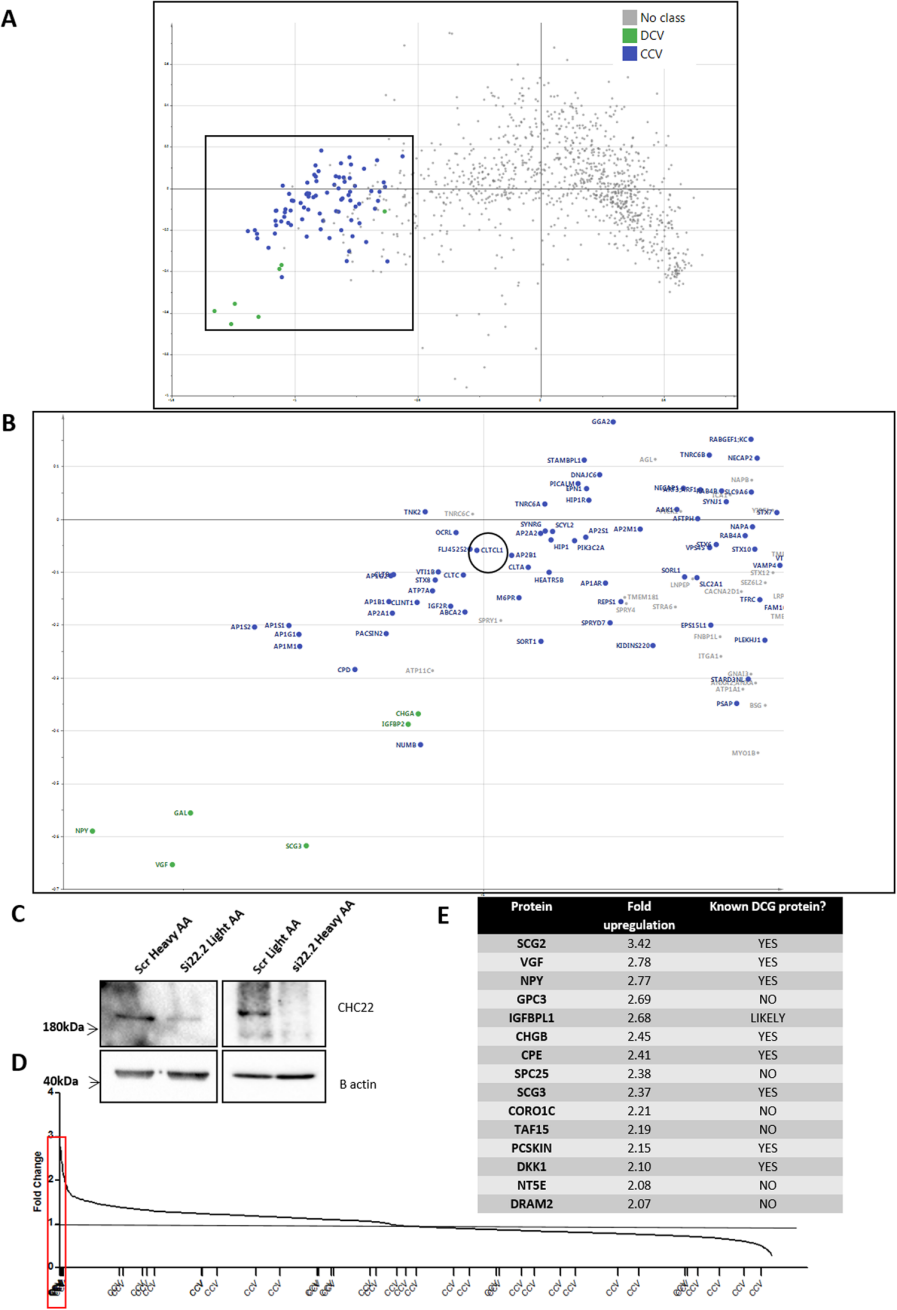
Fractionation profiling to determine the complement of CCVs in SH-SY5Y cells, and cells treated with siRNA to *CLTCL1* show upregulation of dense core vesicle proteins within the vesicle-enriched fraction. (**A**) The spread of all proteins detected in the clathrin coated vesicle (CCV) preparations of SH-SY5Y cells resolved by fractionation profiling. Vesicle enriched membrane fractions were analysed by biochemical fractionation and quantitative mass spectrometry. Protein abundance distribution profiles were subjected to principal component analysis (PCA). Each scatter points correspond to a protein. Established CCV marker proteins (blue) have similar profiles, and cluster separately from other proteins present in the vesicle fraction. Proteins in the same area are hence candidate novel CCV constituents. For full explanation of Fractionation Profiling methodology see Borner *et al*.^14^. (**B**) Magnified view of the proteins resolving as CCV proteins (blue) and DCG’s (green). *CLTCL1* is circled in black and localised in close proximity to *CLTC*. (**C**) Confirmation of knockdown of CHC22 in the lysates of SILAC labelled cells before proteomic analysis. (**D**) Fold change of proteins consistently being up or downregulated in both repeats. CCV proteins are evenly distributed and show no significant change. However, a small subset of proteins were upregulated by more than two fold (boxed in red). (**E**) List of all proteins upregulated more than two fold. Note that 60% of these proteins are known to reside within the dense core secretory granules.

Profiling of clathrin-coated vesicles in SH-SY5Y cells identified ~100 predicted proteins (Supplemental Table 1), many of which had been previously confirmed as *bone fide* CCV proteins, including CHC22 and Sortilin. CHC22 resolved very close to CHC17, demonstrating that it is a component of CCVs in neuronal cells. The analysis also showed a clear separation of CCV proteins from dense core granule (DCG) proteins in SH-SY5Y cells (Fig. 3A and B). We identified 14 neuronal-specific CCV proteins, which are absent from Hela cell CCV preparations^16^ (see Supplemental Table 1).

To determine which components of neuronal CCVs depend on CHC22 and not CHC17, we knocked down CHC22 by siRNA and analysed a vesicle enriched subcellular fraction by SILAC quantitative mass spectrometry (Fig. 3C). Cytoplasmic CHC22 depletion was achieved, sufficient to cause the neuronal differentiation we had previously reported. However, despite efficient knockdown in the cell lysates, and a clear proteomic effect, surprisingly, there was no significant loss of CHC22 from the vesicle preparations themselves. A potential explanation for this is that the residual CHC22 left after knockdown is still incorporated preferentially and efficiently into CCV’s (as previously noted for AP1G1^17^). On vesicle proteome analysis we found no significant changes in CCVs. Nevertheless the vesicle fraction also informs on other types of vesicles, and we found a striking accumulation in a number of DCG proteins - including both canonical DCG structural components such as SCG2 and known DCG cargoes such as NPY, known to be present in embryonic DCGs^18^ (Fig. 3D and E). DCGs are endosomes found exclusively in neuronal cells and specialized secretory cells. Thus our proteomic analysis supports that CHC22 is localised in neuronal CCV’s, but suggests that CHC22 may also function in another CHC17-CCV independent sorting step affecting DCGs.

**Figure 4:**
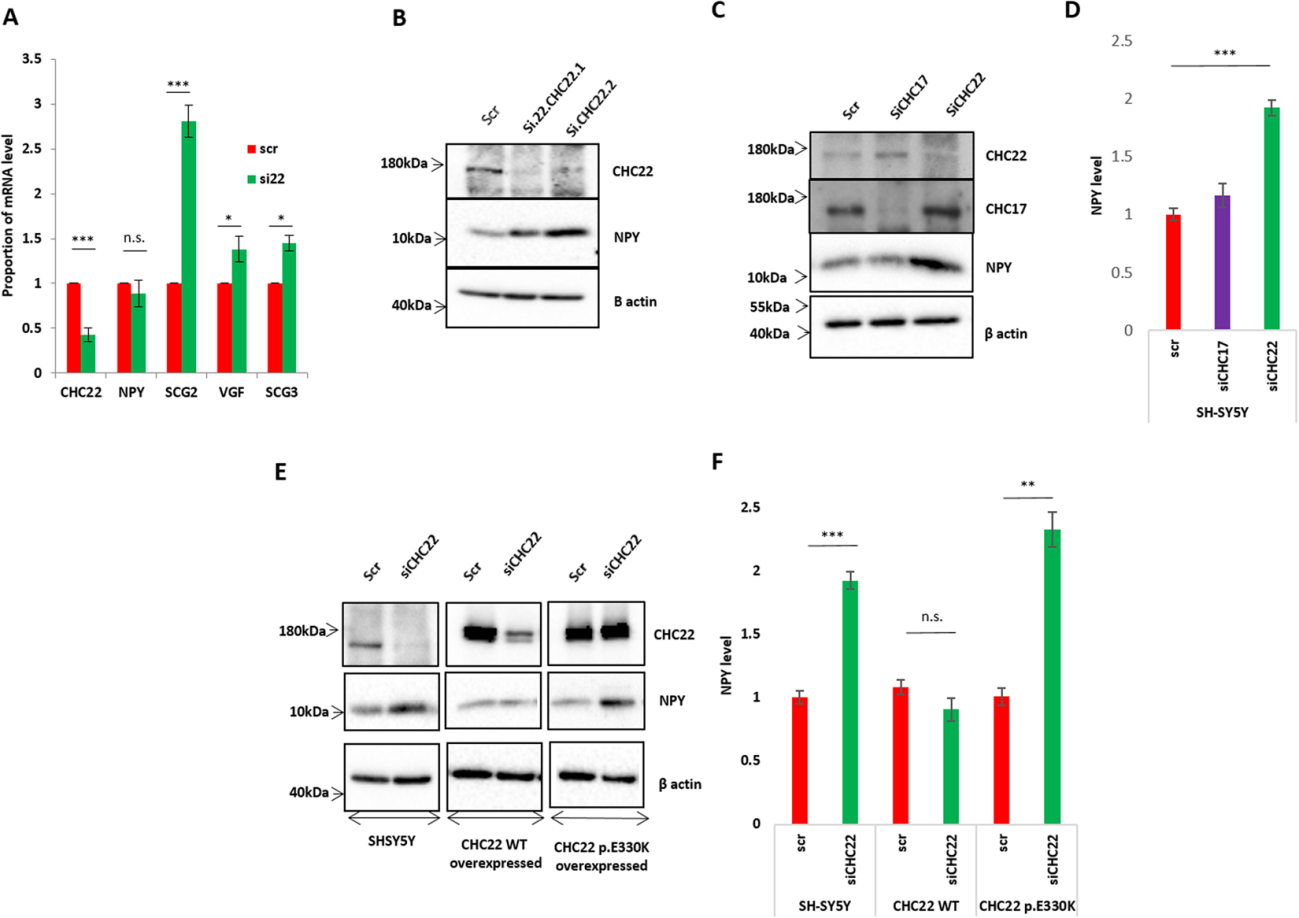
CHC22 loss induces NPY accumulation. (**A**) Quantitative RT PCR analysis of the mRNA levels of NPY, SCG2, VGF and SCG3 in cells treated with CHC22 knockdown, normalised to GAPDH. Note an upregulation of SCG2 and no effect on NPY levels. Statistical tests are one-sample T-test on log transformed proportion data. (**B**) Western blot of whole cell lysates demonstrating knockdown of CHC22 by two different siRNAs caused accumulation of NPY at the protein level (**C**) Knockdown of CHC17 has no effect on NPY levels, whereas CHC22 knockdown induces significant accumulation. (**D**) Quantification of NPY levels from three independent repeats. (**E**) Overexpression of Wild type CHC22, but not mutant p.E330K CHC22 rescues the accumulation of NPY on CHC22 knckdown. Figure complied from three separate blots. (**F**) Quantification of NPY levels from at least three independent repeats. Statistics represent one way ANOVA with Dunnett’s multiple comparison post hoc test (p < 0.05, p < 0.01 p < 0.001 are *, **, *** Respectively).

We determined if DCG protein accumulation was due to upregulation at the mRNA level (indicating a likely secondary effect due to differentiation) or at the protein level (indicating a possible effect on protein trafficking). By use of quantitative RT-PCR analysis we found that *SCG2* was significantly upregulated (3 fold), however, the mRNA levels of the neuropeptide *NPY* remained unchanged, and *VGF*/*SCG3* increased only fractionally (<50%) (Fig. 4A). Despite this, NPY still accumulated in the cell at the protein level (Fig. 4B) (VGF was untestable due to lack of a reliable antibody, but did accumulate 2.78 fold in the vesicle fraction by proteomic analysis. SCG3 accumulated 2.37 fold) (Fig. 3E). We therefore selected NPY as a potential marker of CHC22-mediated transport for further analysis.

### CHC22 controls the trafficking of DCG neuropeptides for lysosomal degradation

**Figure 5:**
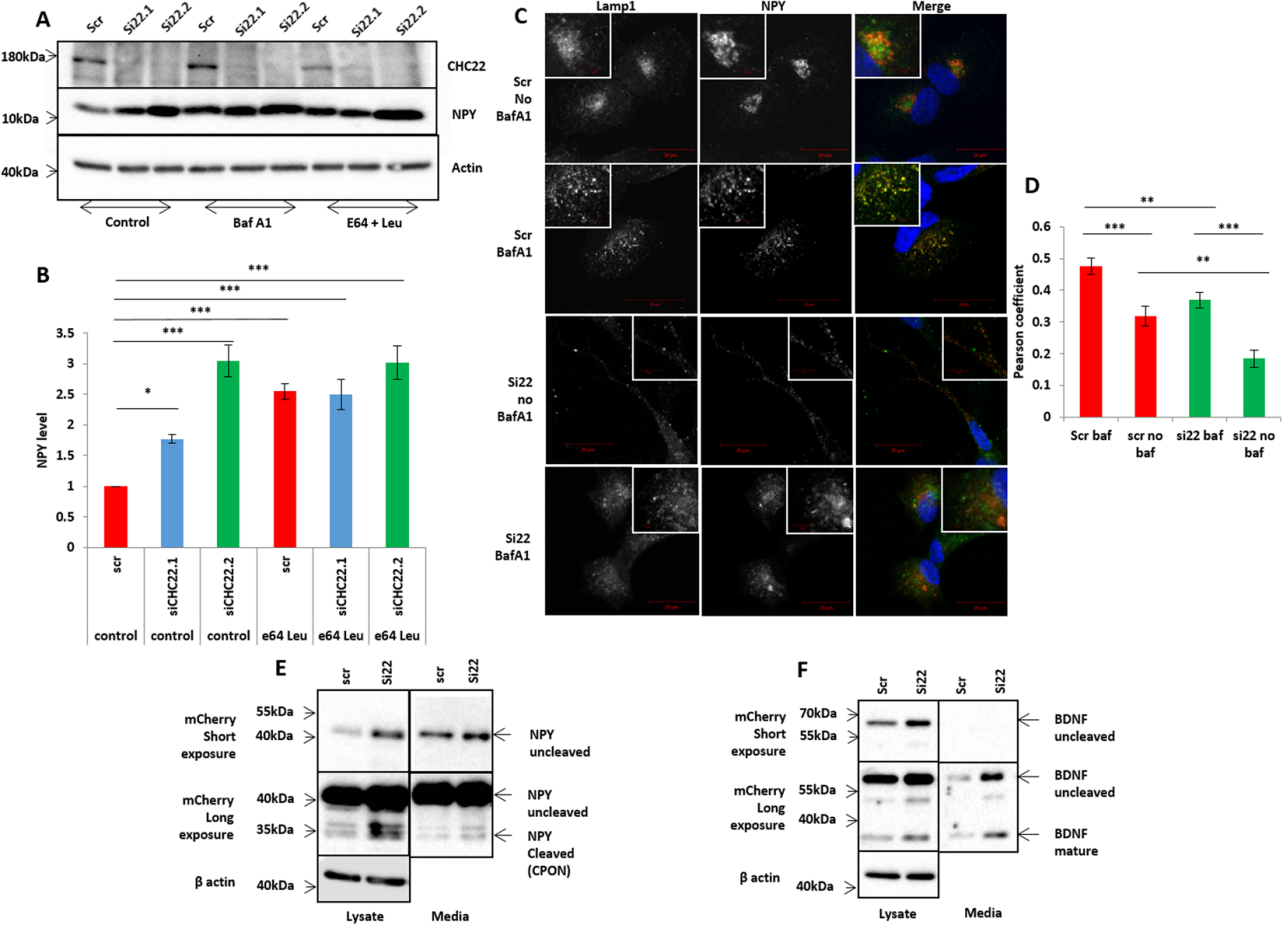
CHC22 affects NPY trafficking to the lysosome. (**A**) Addition of 200 nM BafilomycinA1 (18 hours) or 10 µM E64 and 50 µM Leupeptin for 24 hours resulted in comparable accumulations of NPY in control and knockdown cells. (**B**) Results of densitometry from at least three repeats. Statistics represent one way ANOVA with Dunnett’s multiple comparison post hoc test (p < 0.05, p < 0.001 are *, *** Respectively). (**C**) Immunofluorescence experiments with co-staining for Lamp1 and NPY in the presence or absence of BafA1 and *CLTCL1* knockdown. In untreated cells Lamp1 and NPY show some overlap but rarely clear co-localisation, however after treatment with BafilomycinA1 and a block of endo/lysosome fusion, clear co-localisation is observed. (**D**) Graph representing Pearsons coefficient of co-localisation in each case. At least 24 cells tested for each condition. Together, these results indicate, in the absence of CHC22 NPY is accumulating due to trafficking away from the lysosome and when CHC22 is present, NPY is trafficked (and degraded) within the lysosome. Statistics represent Mann-Whitney U Tests (p < 0.05, p < 0.01, p < 0.001 are *, **, *** Respectively). (**E**) Western blot demonstrating accumulation of NPY in cells with CHC22 knocked down and increased secretion into the media. (**F**) Western blot demonstrating accumulation of BDNF in cells treated with siRNA and notable increases in secretion of both pro and mature BDNF into the media (quantification in supplement Fig. S4). Uncropped blots are in displayed in Supplementary data files.

The role for NPY in pain neuron differentiation is unclear, however NPY receptors are expressed by a significant proportion of PEP1/PEP2 pain sensing neurons^19^, and NPY is trafficked in DCG’s along with other important pain-associated neuropeptides such as BDNF (Supplementary Fig. S2). We asked if lysosomal degradation might control NPY levels in the presence of CHC22. Firstly, we confirmed that accumulation of endogenous NPY in cells treated with siRNA to *CLTCL1* was specific to CHC22 and not clathrin *per se*. Knockdown of CHC22 caused significant accumulation of NPY, yet no effect was observed with knockdown of CHC17 (Fig. 4C and D). This effect was rescued by overexpression of wild type CHC22 but not with mutated CHC22.E330K (the mutation found in a family reported with a congenital lack of pain and touch sensing^7^), nor overexpression of CHC17 (Fig. 4E and F and Supplementary Fig. S10). We then knocked down CHC22 in the presence or absence of lysosomal inhibitors E64 and Leupeptin (which inhibit lysosomal proteolysis^20^), or with BafilomycinA1 (a specific inhibitor of Vacuolar-type H+-ATPase, which blocks endo-lysosomal fusion^21^). Blocking lysosomal degradation by E64 and Leupeptin caused a significant 2.5–3 fold increase in NPY levels (Fig. 5A and B), to levels very similar to those of cells treated with the most effective siRNA against CHC22 (siCHC22.2). Blocking with BafilomycinA1 caused a consistently larger accumulation of NPY, although the magnitude of this effect varied perhaps as a result of broader effects beyond blocking of endo/lysosomal fusion^22,23^ (Fig. 5A, Supplementary Fig. S5). Thus, CHC22 knockdown has the same effects on intracellular NPY levels as treatment with lysosomal inhibitors E64/Leupeptin, or endo-lysosomal fusion inhibitor BafilomycinA1.

In light of this, we sought further evidence that CHC22 promotes neuropeptide degradation through a lysosomal pathway. By confocal microscopy we found NPY to be occasionally colocalised with the lysosomal marker Lamp1 (Supplementary Fig. S2E). Upon treatment of cells with BafilomycinA1 to block endo-lysosomal fusion, this colocalization was significantly increased (Fig. 5C and D). This supports our hypothesis that NPY can be trafficked into endosomes destined for lysosmal fusion. The Lamp1-NPY colocalization was reduced when CHC22 was knocked down, suggesting that CHC22 affects NPY trafficking into endosomes destined for lysosomal fusion. This occurred both in the presence and absence of BafA1, showing that CHC22 reduction alone can divert NPY from being trafficked to lysosomes, enabling neuropeptide secretion.

### CHC22 knockdown increases neuropeptide secretion

To determine if our CHC22 trafficking findings were specific for NPY, or were a group effect on a number of DCG neuropeptides, we chose a second, pain-relevant neuropeptide, BDNF for further study. BDNF is a member of the mammalian neurotrophin protein family that is, similarly to NPY, trafficked in DCGs. BDNF is the ligand for TRKB in the generation of touch sensing neurons, and implicated in neuronal-specific processes including synaptic plasticity^24^. Hence, we decided to test if CHC22 also affected BDNF trafficking, particularly in light of the well-established role of BDNF during neural development^25^ and during SH-SY5Y differentiation^26^.

We have previously shown that CHC22 is acutely downregulated during the induced differentiation of iPSCs into nociceptors, and during Retinoic acid induced SH-SY5Y differentiation; and was essential for differentiation to occur^7^. We here show that this is a paracrine effect. As normal CHC22 levels cause DCG neuropeptide lysosomal degradation, could reduced CHC22 levels allow neuropeptides to be secreted instead of degraded, and explain this paracrine effect? We found NPY and BDNF trafficked into neurites upon knockdown of CHC22 (Supplementary Figs S1 and S3) and asked if this affected their secretion.

When CHC22 was depleted, NPY displayed a significant two fold increase in intracellular levels (both pro- and cleaved forms, and similar to that we had previously seen for endogenous NPY). We also found a significantly increased secretion of the cleaved proNPY product CPON (3 fold) and a trend towards an increase in secretion of un-cleaved NPY (Figs 5E, S4).

The same pattern was observed for BDNF, which accumulated within cells lacking CHC22 expression. The increase was notably more substantial for mature (cleaved) BDNF than proBDNF (uncleaved) (2.4 fold increase, and 1.3 fold increase respectively) hence suggesting CHC22 might directly or indirectly affect the cleavage of proBDNF within the cell. Both proBDNF and mature BDNF displayed significantly increased secretion into the media (2 and 3 fold respectively) (Figs 5F, S4).

Using FRAP (Fluorescence Recovery After Photobleaching) we also found NPY and BDNF are predominantly trafficked in an anterograde direction and are secreted from neurites (Supplementary Fig. S3). Together, this suggests that CHC22 depletion allows increased neuropeptide secretion from neurites.

## Discussion

Previous work on human painless patients with *CLTCL1* mutations and this proteomic analysis of CHC22 in neuronal cells suggest a novel developmental role for CHC22 in regulating sensory neuron differentiation in the human peripheral nervous system.

As CHC22 makes up approximately 1% of the total clathrin heavy chain in the cell (the remainder being clathrin heavy chain 17, CHC17), it is surprising that mutation or knockdown of CHC22 protein is sufficient to induce a phenotype. However, we found that only expression of CHC22, and not CHC17, can rescue the neuronal differentiation phenotype and NPY accumulation induced by CHC22 knockdown. This result confirmed that CHC22 has a specific role in neuronal differentiation. This is interesting in light of the recent report that CHC22 forms distinct coated vesicles to that of CHC17^27^.

We found that the CHC22-dependent stage of neural differentiation is mediated through a paracrine effect, and likely also an autocrine effect. Previously we had shown that the neuronal differentiation could be induced by knock-down of *CLTCL1* or the addition of Retinoic acid, but that both were reliant on a reduction in CHC22 levels^6^. The mechanism described in this paper suggests that CHC22 controls the secretion of neuropeptides which drive differentiation, and that it is a reduction in the levels of CHC22 that leads to the initial commencement of neuropeptide section. CHC22 level reduction hence licences a neuronal developmental step – and in an individual cell this would be a CHC22 autocrine effect. However, in the induction of differentiation in cells (with unaltered CHC22 levels) treated with media from *CLTCL1* knock-down cells (which had significant CHC22 level reduction and then had undergone differentiation) it is a paracrine event. And this event is induced by secretions from the differentiating cells in which CHC22 has been downregulated. We conclude that the mechanism by which CHC22 causes the differentiation phenotype we observed is by controlling secretion of trophic neuropeptides.

CHC22 has been implicated in endosomal trafficking, possibly in conjunction with CCVs, but CHC17 independent roles have also been postulated^5,6,13^. We used fractionation profiling to perform an unbiased proteomic analysis of a subcellular fraction highly enriched in vesicles and found that CHC22 had the same profile as CHC17 CCVs. The composition of CHC17-associated CCVs was largely unaffected by CHC22 knockdown, but a small number of non-CCV proteins accumulated significantly. Strikingly 60% of these were known to reside in dense core granules (DCGs). These are neuronal-specific vesicles (partially coated with clathrin during their maturation) defined by specific structural components (including granin proteins, e.g. SCG2 and SCG3) and cargoes (including neuropeptides e.g. NPY, BDNF and VGF)^28^. Hence this data suggests a neuronal function that is specific to CHC22 and which is independent of CHC17. We demonstrate that in the presence of CHC22, DCG-localised neuropeptides are trafficked to the lysosome, whereas when CHC22 is depleted they are instead secreted sufficiently to cause autocrine/paracrine differentiation *in vitro*. This accords with a previous report for the neuropeptide BDNF, where it is synthesised in excess but targeted to lysosomes for degradation rather than secreted, until a specific cue is received^20^. Although our data suggests CHC22 alters the trafficking of neuropeptides directly, the possibility that CHC22 might have functions outside trafficking which lead to differentiation and altered sorting of neuropeptides, cannot be excluded and is an area for further work.

We propose that temporal control of CHC22 levels *in vivo* is essential for triggering sensory neural precursor differentiation. Consistent with this, there is a failure of touch and nociceptive in *CLTCL1* human mutants. We suggest that this may be caused by premature differentiation of sensory neural precursors, and as these cells are migrating from the neural crest to the dorsal root ganglia during this developmental time period, they would be in premature ectopic positions potentially preventing normal trophic and axonal guidance effects.

While the exact repertoire of neuropeptides required for pain and touch neuron differentiation is unknown, our results suggest their trafficking is non-redundantly influenced by CHC22. In our experimental conditions CHC22 showed the greatest effects on the neuropeptides SCG2, VGF, NPY and IGFBPL1. However, further study should aim to elucidate which peptides are responsible for sensory neuron differentiation in human primary neuronal models, and the precise functional role for CHC22 in their control.

## Materials and Methods

### Cell lines and culture conditions

SH-SY5Y cells were purchased from Sigma Aldrich, and were cultured in 100% Dulbecco’s modified Eagle medium (DMEM) supplemented with 10% FBS, 100 U/ml penicillin-streptomycin (pen-strep) at 37 °C and 5% CO_2_.

For SILAC labelling of SH-SY5Y cells, the cells were passaged for >14 days in SILAC medium (Thermo-Fisher Scientific) supplemented with 10% (vol/vol) dialyzed foetal calf serum (10,000 MW cut-off, Gibco) and “heavy” amino acids (L-arginine-^13^C_6_^15^N_4_:HCl [50 mg/litre] and L-lysine-^13^C_6_^15^N_2_:2HCl [100 mg/liter]; Cambridge Isotope Laboratories), supplemented with 100 U/ml penicillin-streptomycin, or in the same component SILAC media except with “light” amino acids (L-Arginine:HCl and L-Lysine:HCl, Sigma Aldrich).

To create stably expressing clonal cell lines of CHC22.GFP and CHC17.GFP, the method used was the same as described previously in (Nahorski *et al*.)^7^.

### DNA constructs, antibodies and RNA interference

All “Silencer select” siRNA oligos were purchased from Life Technologies. Transfection of siRNA was achieved using Lipofectamine RNAimax Transfection Reagent (ThermoFisher Scientific) according to the manufacturer’s instructions for a reverse transfection. Briefly, for 4 days, 1 hit transfection cells were plated at 1.5 × 10^4^ cells/ml onto a mixture of 15 nM siRNA diluted with 200ul Optimem media and 6.5 ul RNAimax reagent, and then incubated for 4 days. For 2 hits, 5 days transfection media was changed on day 3 and a second hit of siRNA added. For proteomic analysis cells were plated at 8.5 × 10^6^ cells per plate (245mm^2^, Corning) and treated with 1 hit siRNA for 4 days, at which point cells were 95% confluent.

Primary antibodies used were Human Clathrin Heavy Chain 2/CHC22 Polyclonal Sheep IgG Antibody (R&D Systems), Anti-beta actin (AC-15) antibody (ab6276) (Abcam, Cambridge UK), Neuropeptide Y (D7Y5A) XP Rabbit mAb (Cell Signalling), Anti GFP (Living Colors A.v. Monoclonal Antibody jl-8, Clontech), Anti-mCherry rat Monoclonal Antibody (16D7, ThermoFisher Scientific), Monoclonal ANTI-FLAG M2 antibody, mouse monoclonal (Sigma-Aldrich), Ant Sortilin (612100 BD Transduction Laboratory), Anti-TrkB mouse antibody (610101 BD Transduction Laboratory), Anti-KIDINS220 (ab3490, Abcam), Anti- Anti-LAMP1 (ab25630, Abcam), Anti-Chromogranin C (SCG2) antibody (ab12241, Abcam), Anti-βIII-Tubulin (T8660, Sigma).

siRNAs were purchased from Life Technologies or Qiagen. The siRNAs against CHC22 were GGCYCAAUCGUGAACUUCAtt (siCHC22.1) and GAAGAUGUUUGAUGACAUtt (siCHC22.2). Scrambled control was UUCUCCGAACGUGUCACGUtt. When not described explicitly, siCHC22.2 was used as the most effective RNAi available.

To achieve plasmid constructs of NPY and BDNF where the pro and mature forms could be identified and quantified by western blot and visualised by confocal microscopic analysis, both ORF’s were synthesised with a FLAG tag sitting after the N-terminal signal peptide. These constructs were then sub-cloned into the pmCherry-N1 Vector (Clontech) to add an mCherry tag to the C-terminus. These constructs were transfected into SH-SY5Y cells using the X-tremeGENE HP DNA transfection reagent according to the manufacturer’s recommendations (4 µg DNA added to 6 ul XtremegeneHP diluted in 200 µl Optimem per well of a 6 well plate).

### Neurite outgrowth assays

Neurite outgrowth was carried out as previous described^7^. For the CHC17 and CHC22 over expressing cell lines, only cells expressing GFP were quantified.

To test the effects of secretions collected from control and CHC22 knock-down cells on neurite outgrowth of fresh cells, initially 6 well plates of cells were treated with siRNA as described above and left for 3 or 4 days. On sequential days further siRNA experiments were set up so that there were always fresh siRNA-treated cells available for collection of media throughout the 7 day experiment. On day 3, a fresh plate of SH-SY5Y cells was plated into a 6 well dish at 1.5 × 10^4^ cells/ml. Also on day 3, the media was replaced on the siRNA treated cells with 1 ml of Optimem media and left for 12 hours to collect secretions. On the morning of day 4, the secretions were collected and concentrated using Amicon Ultra – 0.5 mL Centrifugal Filters (Millipore), and the cells lysed for confirmation of successful knockdown. The concentrated secretions were then added to the fresh cells. This process was repeated for a further 3 days, so that the “fresh” cells had been treated with 3 days of secretion collections. At this time point the cells were imaged using EVOS FLCell Imaging System and the neurite length blinded and quantified as previously.

### Protein extraction

Cells were grown in 6-well plates or 10 cm^2^ dishes, washed with phosphate buffered saline (PBS) and scraped in RIPA buffer (Tris, pH 7.4, NaCl 150 mM, ethylenediaminetetraacetic acid (EDTA) 0.5 mM, 1% Triton), containing protease inhibitors (Roche Applied Sciences). Lysates were cleared by centrifugation at (13,300 × g at 4 °C for 20 minutes) and levels of total cellular protein tested using the DC protein assay kit according to manufacturer’s instructions (BioRad Laboratories, Ltd).

### Protein blotting

Total cellular protein was separated using either 8% or 15% SDS-page gels, NuPAGE Novex 3–8% Tris-Acetate Protein Gels or NuPAGE 4–12% Bis-Tris Protein Gels. Primary antibodies are described above. Secondary antibodies were purchased from Dako and signal detected using the enhanced chemiluminescence (ECL, Amersham) western blot analysis system. Blots were analysed using a Chemidoc MP imaging system, and densitometry carried out using ImageJ.

### Confocal Microscopy

Primary antibodies used for immunofluorescence are given above. Cells were cultured on poly-L Lysine coated coverslips and fixed by 10 minutes incubation in 4% paraformaldehyde. Cells were permeabilised by 10 minutes incubation in 0.3% tritonX100 solution and blocked with 30 minute, room temperature incubation in 5% BSA. Fixed cells were then stained with primary antibodies for 1 hour in 5% BSA and fluorescent secondary antibody also for 1 hour. Secondary antibodies used were Alexa Fluoro 546 goat anti-rat, Alexa Fluoro 546 goat anti-rabbit, Alexa Fluoro 546 donkey anti-mouse, Alexa Fluoro 633 goat anti-rabbit (All from Life Technologies). Coverslips were mounted onto glass slides using Prolong Diamond Antifade Mountant with DAPI (Molecular Probes). Cells were visualised with a LSM710 or LSM780 confocal microscope.

### Clathrin Coated Vesicle Preparations

Preparation of cell lysates was done at 4 °C. SH-SY5Y cells were grown to confluence and washed once in ice cold phosphate-buffered saline (PBS) and once in ice-cold MES buffer ((0.1 M 2-(N-morpholino)ethanesulfonic acid [MES], pH 6.5 [adjusted with NaOH], 0.2 mM ethylene glycol tetraacetic acid, and 0.5 mM MgCl_2_). Cells were scraped into 6.5 ml of MES buffer and lysed in a 30 ml Potter-Elvehjem homogenizer with 20 strokes of a motorised pestle, before being forced 10 times through a 0.8 mm needle and once through a 0.5 mm needle. The method used for HeLa cells was then followed (Borner *et al*.^16^). For proteomic analysis, CCV enriched fractions were prepared from SH-SY5Y cells labelled with SILAC heavy (control) or light amino acids (CHC22 knockdown), in biological duplicate. Equal quantities of CCV fractions from control and CHC22 knock-down cells were mixed, and analysed by mass spectrometry.

### Fractionation profiling

The clathrin-coated vesicle (CCV) proteome from SH-SY5Y cells was determined by Fractionation Profiling, as previously described in the paper “Fractionation profiling: a fast and versatile approach for mapping vesicle proteomes and protein-protein interactions”. (Borner *et al*.^14^). Principal component analysis was performed in SIMCA 14 software (Umetrics).

### Mass spectrometry

Protein samples were subjected to in-gel (Borner *et al*.^14^), or in-solution digest and SDB-RPS peptide fractionation^29^, as described. For fractionation profiling, mass spectrometry was performed with a Q Exactive instrument (Thermo Fisher Scientific, Germany) as described in Borner *et al*.^16^; for analysis of CCV fractions, a Q Exactive HF (Thermo Fisher Scientific, Germany) was used, as reported in (Itzhak *et al*.)^29^. Raw files were processed with MaxQuant software^30^, and analysed as described (Borner *et al*.,^14,16^).

### Quantitative RT-PCR

mRNA was extracted from cells using the RNAeasy Plus Mini Kit (Qiagen) according to the manufacturer’s instructions. The concentration of the mRNA was calculated and 1 µg converted to cDNA using the qScript cDNA synthesis kit (Quanta Biosciences). Levels of *CLTCL1, NPY, SCG2, VGF, SCG3* and *GAPDH* were assayed using pre-designed TaqMan Gene Expression Assays (ThermoFisher Scientific). The level of gene expression was normalised to that of GAPDH for each sample assessed.

### Basal secretion assays

SH-SY5Y cells were seeded and siRNA treated as described previously with a 2 hits, 5 days protocol (2^nd^ hit on day 4). On the third day, either the mCherry/Flag NPY or BDNF constructs were transfected using X-tremeGENE HP DNA transfection reagent. One plate was harvested to confirm knockdown of CHC22 at day 5 by quantitative RT-PCR (results only included if knockdown of over 55% achieved). On day 5, media was replaced with 1 ml of serum free media and the secretions collected for 4 hours. The media was then concentrated using Amicon Ultra – 0.5 ml Centrifugal Filters (Millipore) and the cells lysed. The levels of pro and mature neuropeptide in the lysates and collected secretions were analysed by western blot and densitometry (ImageJ). The experiments were repeated a minimum of five independent times.

### Lysosomal blocking experiments

To test for trafficking of endogenous NPY to the lysosome, two different methods for blocking lysosomal degradation were used. After 3 days of knockdown, media was replaced with media containing either Bafilomycin A1 (Alfa Aesar) at 200 nM concentration for 18 hours, or media containing 10 µM E64 and 50 µM Leupeptin (both Sigma Aldrich) for 24 hours. After this time, cells were lysed and the levels of endogenous NPY quantified by western blot. For co-localisation analyses of NPY with Lamp1 in the presence of Bafilomycin A1 the experiment was carried out as above with cells on coverslips and 18 hours incubation in BafA1.

### Confocal microscopy using FRAP to track Neuropeptide trafficking in neurites

For live cell imaging, cells were plated onto glass coverslips and imaged using an LSM780. Movies were made of at least 100 frames. For adapted FRAP analysis, after 3 frames, a consistent section of neurite was bleached and a further 97 frames of movie recorded. The movies were analysed using Volocity software. Individual peptides were tracked frame by frame until they had moved out of the originally bleached region. Over the 100 frames, every peptide moving into the region was tracked until the region became too full of vesicles that accuracy became impossible. The speed and direction of travel of the vesicles (anterograde away from the cell body or retrograde towards the cell body) were recorded and compared.

### Data Availability

The datasets generated during and/or analysed during the current study are in Table S1 or are available from the corresponding author on reasonable request.

## Electronic supplementary material

Supplementary Information

Supplementary Table 1
