# Supplementary material for: Clathrin heavy chain 22 contributes to the control of neuropeptide degradation and secretion during neuronal development
Source: Sci Rep. 2018 Feb 5;8:2340. doi: 10.1038/s41598-018-19980-0 (PMC5799199; doi:10.1038/s41598-018-19980-0)

**Clathrin heavy chain 22 contributes to the control of neuropeptide degradation and secretion during neuronal development - Supplementary Information**

**Authors:**

Michael S. Nahorski^1*^, Georg HH Borner^2^, Samiha S Shaikh^1^, Alexandra K Davies^1^, Lihadh Al-Gazali^3^, Robin Antrobus^1^, C Geoffrey Woods^1*^

Affiliations:

1. Cambridge Institute for Medical Research, University of Cambridge, Cambridge, CB2 0XY, UK.

2. Max Planck Institute of Biochemistry, Department of Proteomics and Signal Transduction, Am Klopferspitz 18, 82152 Martinsried, Germany.

3. Department of Peadiatrics, College of Medicine and Health Sciences, United Arab Emirates University. P.O.Box 17666, Al-Ain, United Arab Emirates l.algazali@uaeu.ac.ae

**Corresponding authors*:**

MSN Tel +44123 336 143, Fax +441223 762 323, Email: [msn27@cam.ac.uk](mailto:msn27@cam.ac.uk).

CGW Tel +44123 336 143, Email: cw347@cam.ac.uk

**Number of Figures and Tables:** 4

**Conflict of Interest:** The authors declare no competing financial interests

**CCV protein predictions SH-SY5Y cells**

Supplementary table 1 – uploaded separately.

**NPY subcellular localisation changes with CHC22 level reduction**

We used confocal microscopy to assess the localization of endogenous NPY. In cells expressing endogenous CHC22, NPY was found in discrete foci mainly in the perinuclear compartment (57%) in the parental cells (tightly co-localized with the Trans-Golgi-Network (Figure S1 A); upon onset of differentiation in CHC22 knockdown cells NPY became also localized within newly formed neurites (Figure S1 B).

**Figure S1- NPY cellular localisation**


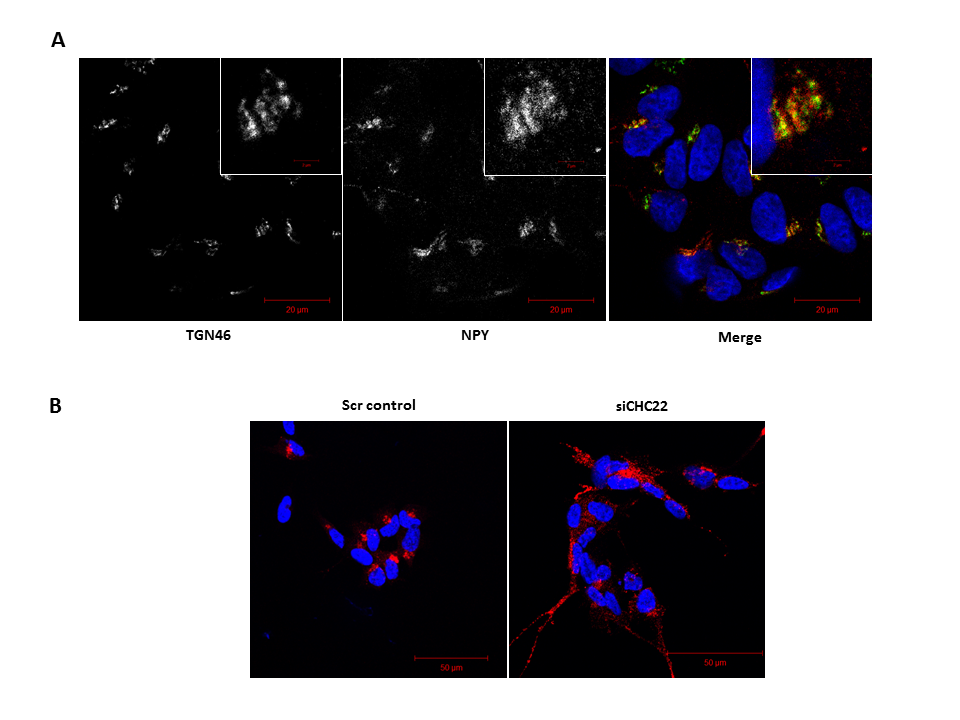


**Characterisation of BDNF and NPY tagged constructs**

BDNF and NPY were each tagged at the N-terminus (after the signal peptide cleavage site) with flag and at the C-terminus with mCherry, allowing direct visualisation of both the cleaved mature neuropeptide (mCherry signal) and the un-cleaved larger pro-peptide (co-incident mCherry and flag signal) by both immunofluorescence and western analysis (Figure S2 A). We confirmed that both NPY and BDNF constructs showed the expected vesicular localization. Both pro-NPY and mature-NPY localized in the same vesicles (Figure S2 B) and showed significant colocalization between NPY and SCG2 (a constituent protein of dense core granules) at the trans-Golgi-network (TGN) and occasionally at the cell periphery (Figure S2 C. BDNF localized to SCG2 positive vesicles at the TGN and throughout the cytoplasm (Figure S2 D).

There was no significant colocalization with early or late endosomes (EEA1/VPS35) and only limited colocalization with endo-lysosomes (Lamp1) (Figure S2 E).

**Figure S2- Characterisation of location/identity of BDNF and NPY tagged constructs**


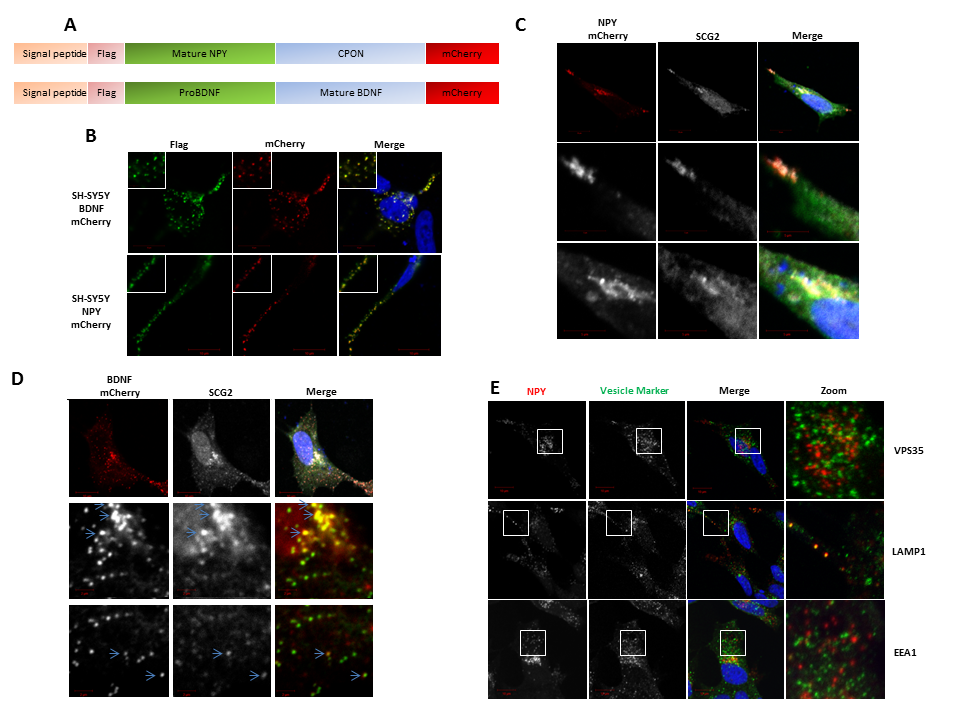


**CHC22 loss lead to DCG neuropeptide transport in neurites and secretion**

To discover if NPY and BDNF could be being secreted from neurites, we measured the direction of trafficking of BDNF and NPY labelled dense core granules by live cell imaging. Both neuropeptides were trafficked in both anterograde and retrograde directions. To quantify the proportions of vesicles moving in each direction, we adapted the FRAP (Fluorescence Recovery After Photobleaching) technique to bleach a region of neurite. We then tracked each neuropeptide across the distance of the bleached area using Volocity software. For both NPY and BDNF, the significant majority of vesicles trafficked in an anterograde direction away from the cell body (Sup Fig 3A, B, C) suggesting a proportion are being secreted from the neurite ends. (There was no significant difference in speed of the vesicles depending on direction, although speed was variable) This data is in agreement with the increased levels of neuropeptide found in the media of cells in which CHC22 is depleted and suggests that CHC22 depletion leads to increased neuropeptide secretion from neurites.

**Figure S3- Live cell imaging of NPY and BDNF transport in CHC22 knocked down cells**


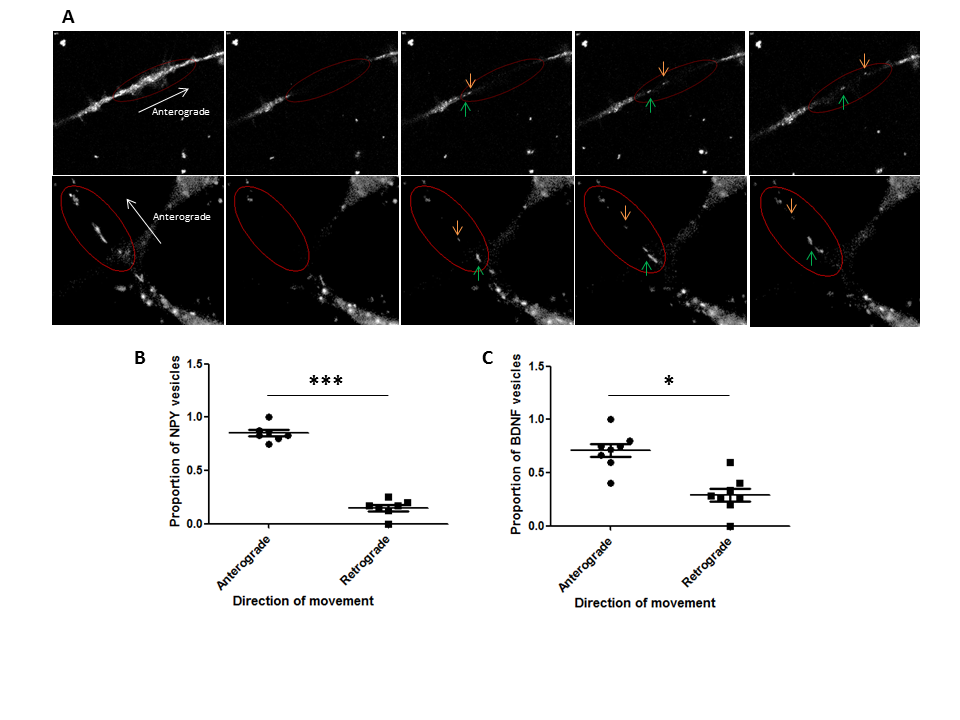


**S3 Legend**

1. Example screenshots of neuropeptide trafficking. Note bleaching in the second images allows the tracking of individual neuropeptides in subsequent images. Different coloured arrows highlight the same vesicle in each cell over time. (b) and (c) are graphs of quantification of the proportion of cells moving in either an anterograde (away from cell body) or retrograde (towards cell body) direction for NPY and BDNF. Each point on the graph represents the proportion for one cell. Significance tested by calculating the anterograde over retrograde ratio, log-transforming the data and testing by one tailed T Test vs 0. One star represents p<0.05, three stars p<0.001. A total of 94 vesicles were tracked from 14 cells from 3 independent experiments.

**Fig S4 – Quantification of NPY and BDNF secretion (from Figure 4 H and I)**


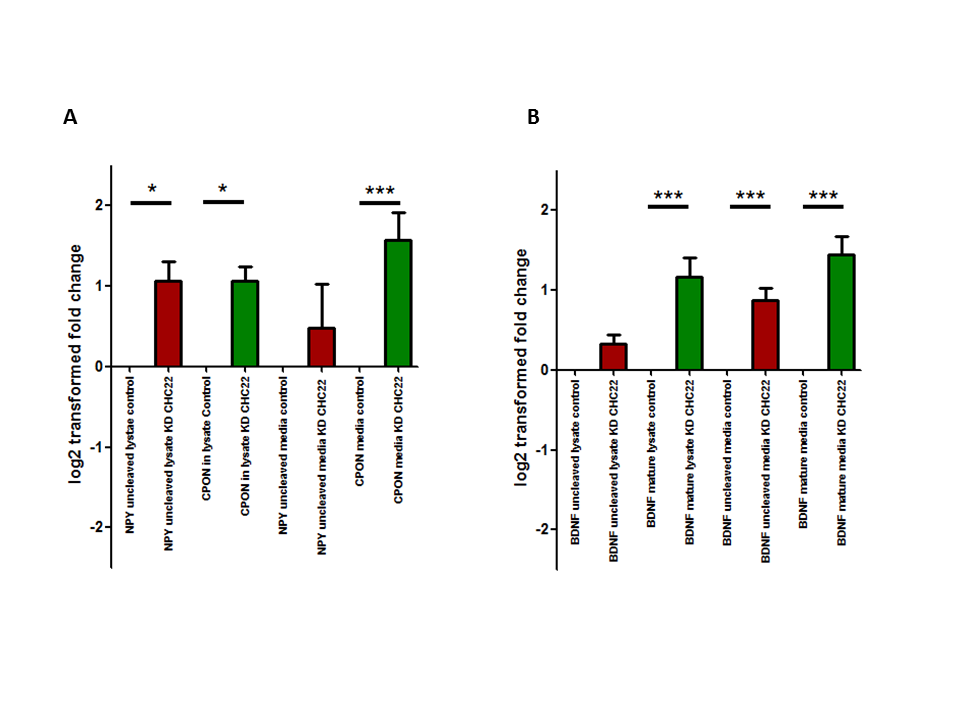


Fig S4 Legend

Quantification of transfected neuropeptide secretion by densitometry of at least four independent experiments for NPY (A) and BDNF (B). Statistical tests were ANOVA on log2 transformed data, with Bonferroni corrected post hoc tests. * represents p<0.05, *** p<0.001. Representative blots are given in Figure 4 H and I.

**Figure S5 - Quantification of Neuropeptide Y accumulation with Bafilomycin Treatment in cells treated with siRNA to CLTCL1**


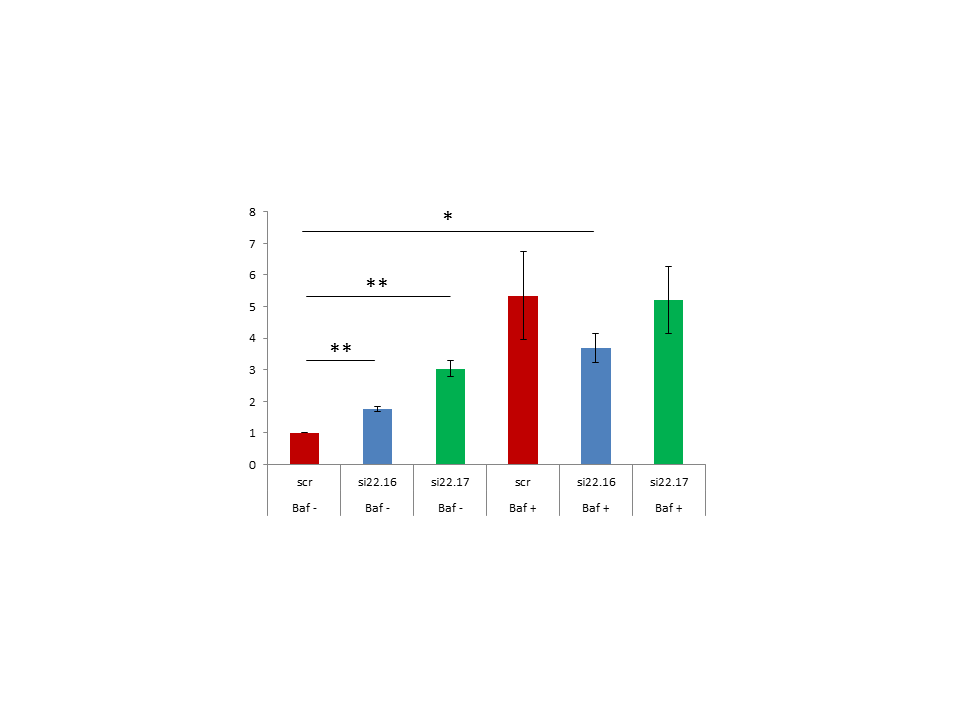


**Fig S5 legend**

Results of densitometry from at least three repeats. Statistics represent one way ANOVA (P<0.0001) with Dunnett’s multiple comparison post hoc test (p<0.05, p<0.01 are *, ** respectively).

**Figure S6 – CHC22 Knock down in clathrin overexpressing cell lines**

**
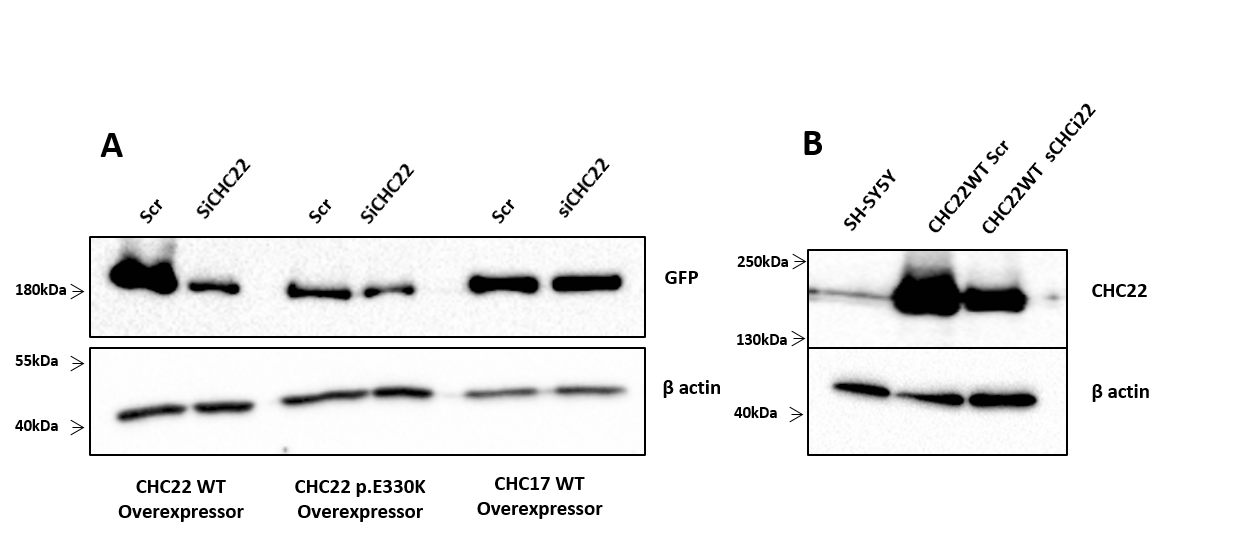
**

1. Levels of GFP tagged CHC22 or CHC17 in cells treated with either scrambled control or siRNA targeted against endogenous CHC22. B. CHC22 levels of CHC22 WT overexpressing cells (CHC22 WT.A) after control of CHC22 knock down compared with endogenous levels of CHC22 in SH-SY5Y cells

**Figure S7 – GFP-tagged Clathrin overexpression in stable SH-SY5Y cell lines**

**
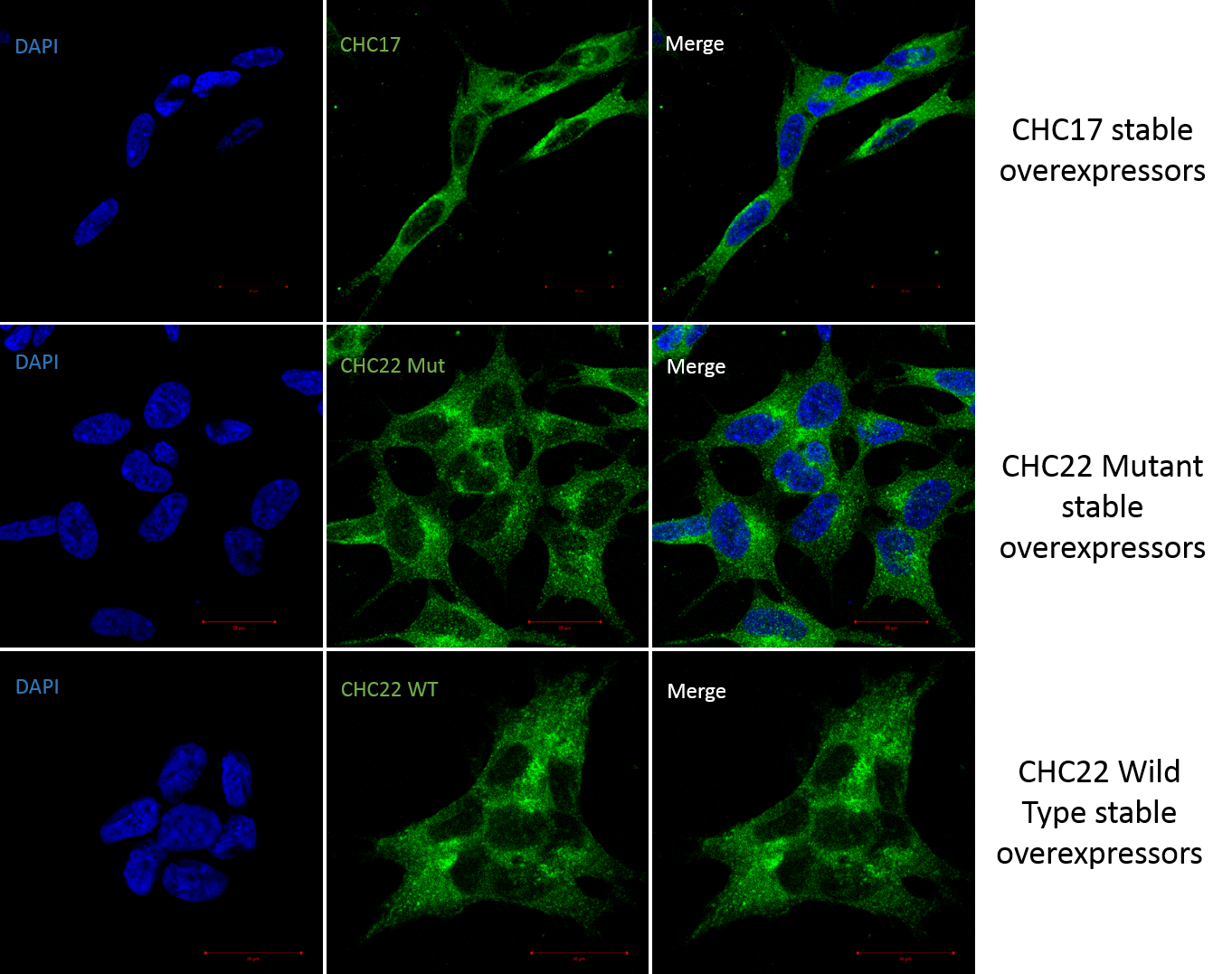
**

**Figure S8 – Clathrin overexpression in stable SH-SY5Y cell lines**


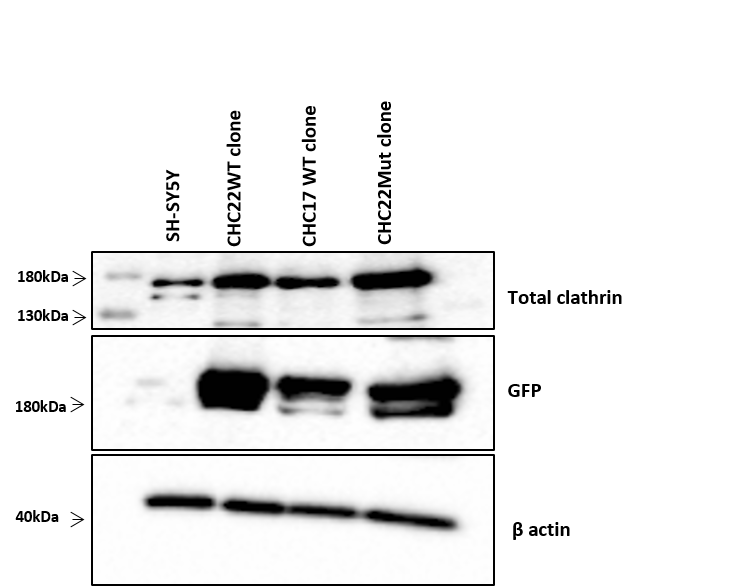


Western blot demonstrating the comparative levels of total clathrin (using CHC17 x22 antibody) and GFP-tagged overexpressed clathrin CHC22 and CHC17 in the overexpressing cell lines used in this study.

**Figure S9 – Biochemical marker of differentiation upon CHC22 knock down**

**
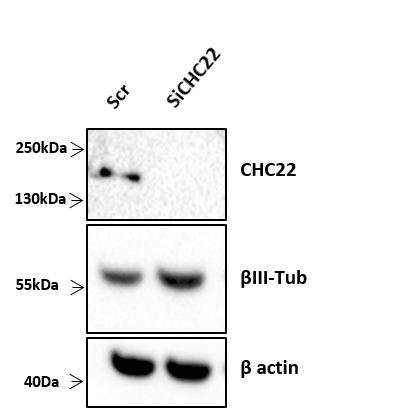
**

Western blot demonstrating increased levels of βIII-tubulin expression as SH-SY5Y cells undergo differentiation after 5 days of CHC22 knock down (2 hit).

**Figure S10 – CHC17 overexpression does not rescue NPY accumulation**


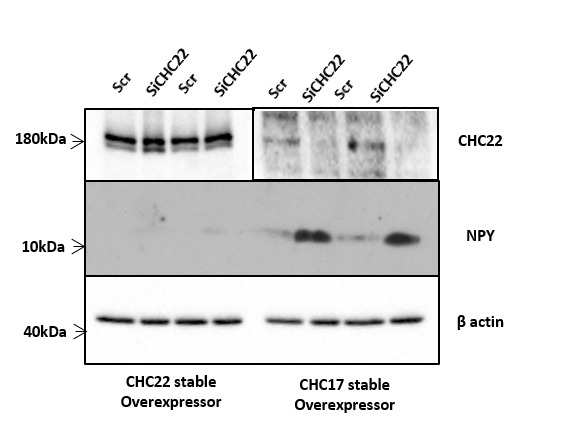


The siRNA resistant CHC22 overexpressing cell line (CHC22WT.B) above is the same as was used in Nahorski *et al* 2015. Knockdown of endogenous CHC22 in these cells does not cause accumulation of NPY. However, stable overexpression of CHC17 does not rescue NPY accumulation upon CHC22 knockdown. Two repeats are shown for each cell line. The CHC22 panel are from the same blot, but two different exposure times due to the high levels of CHC22 in the CHC22 stable overexpressors compared with endogenous CHC22 in the CHC17 overexpressors.

**Uncropped Blots**


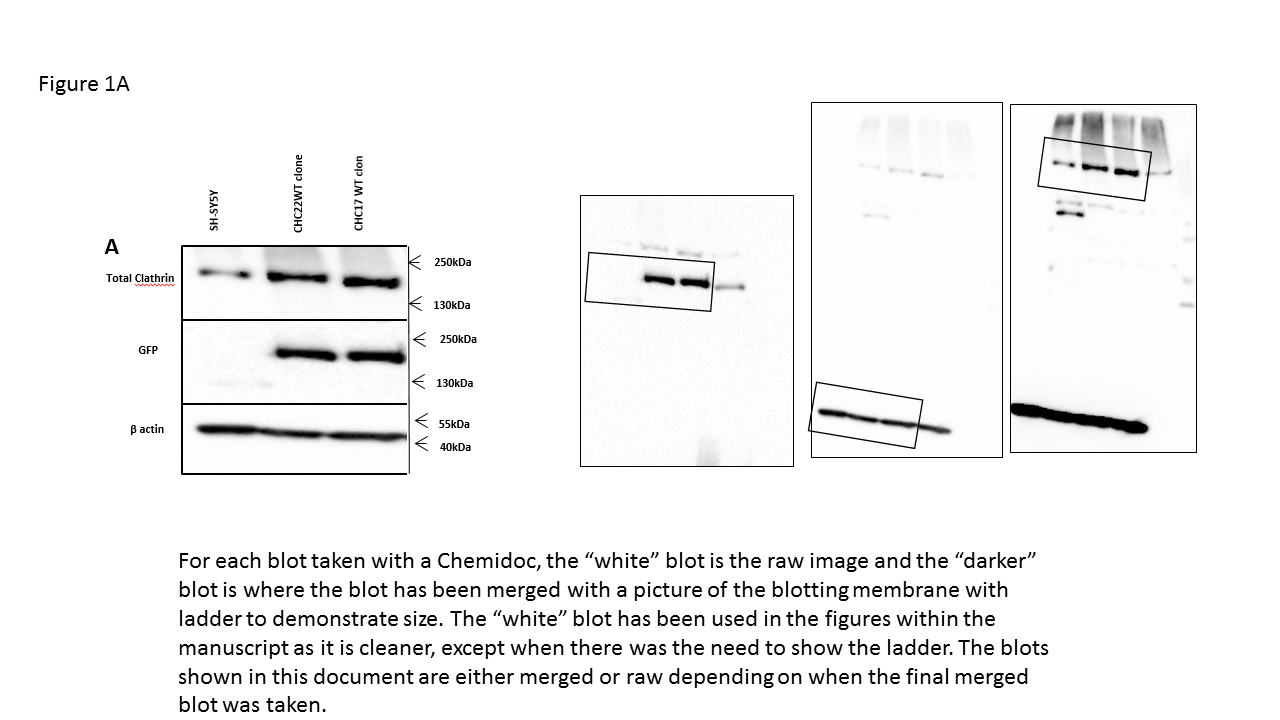


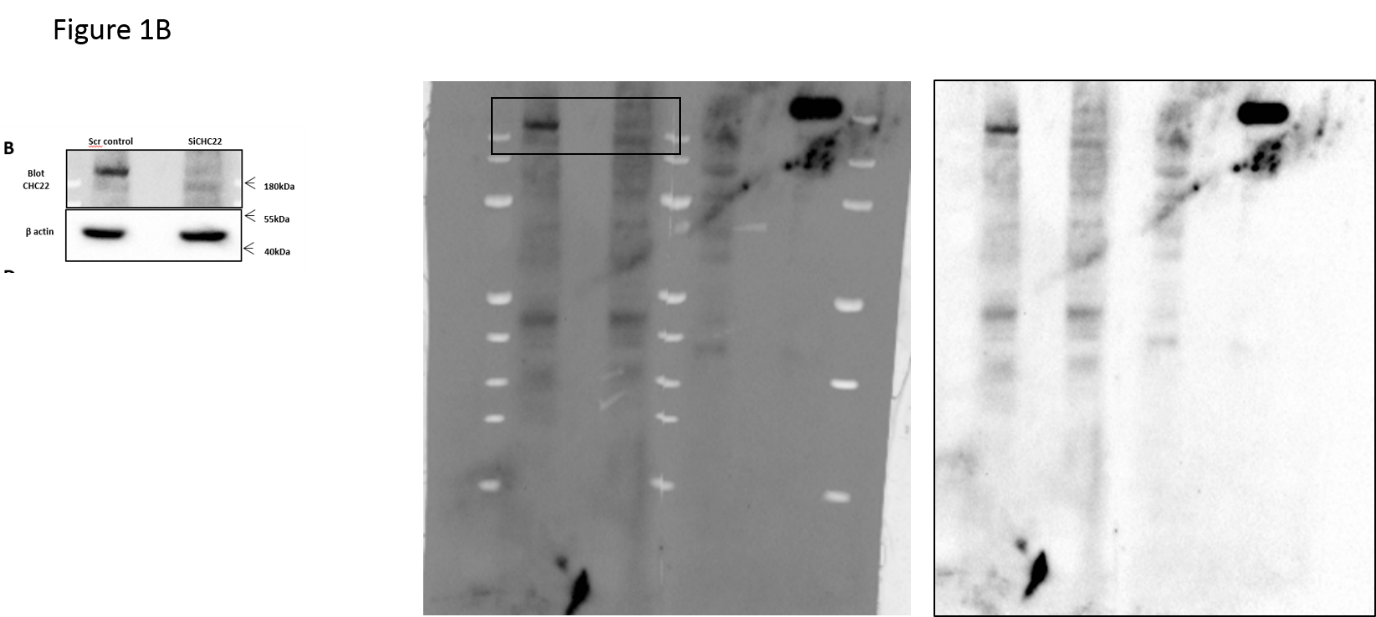


**
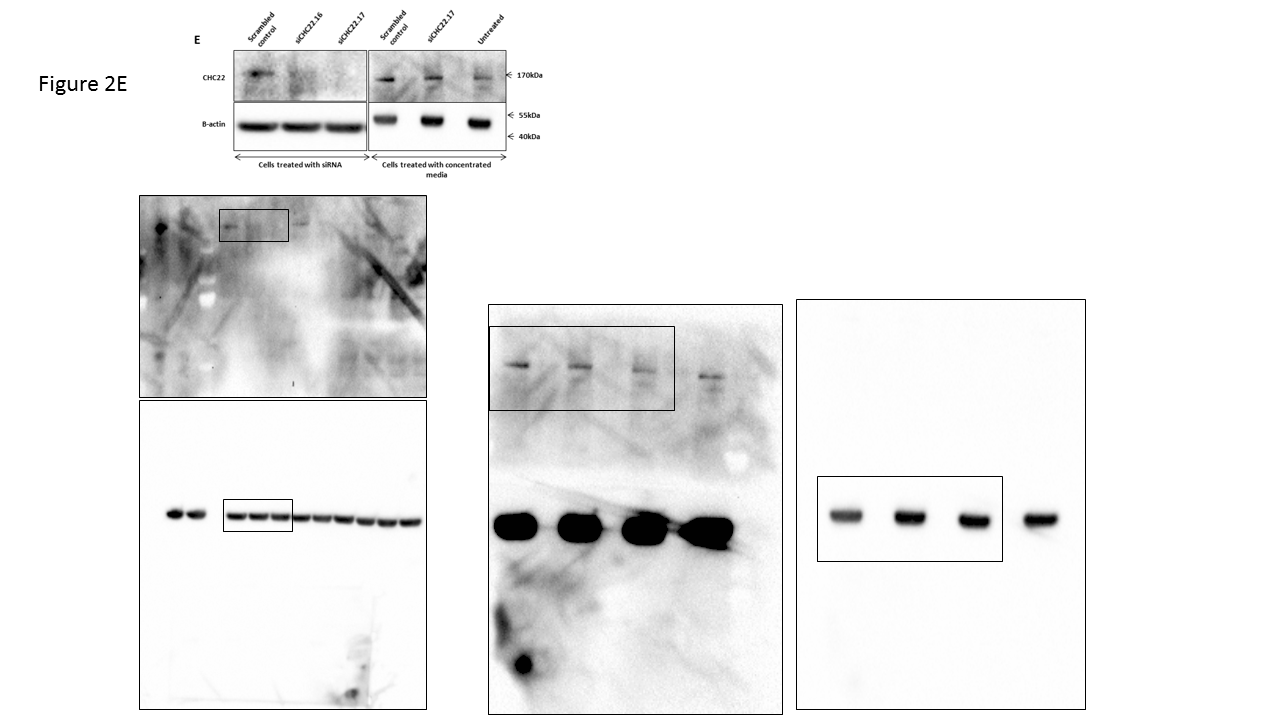
**


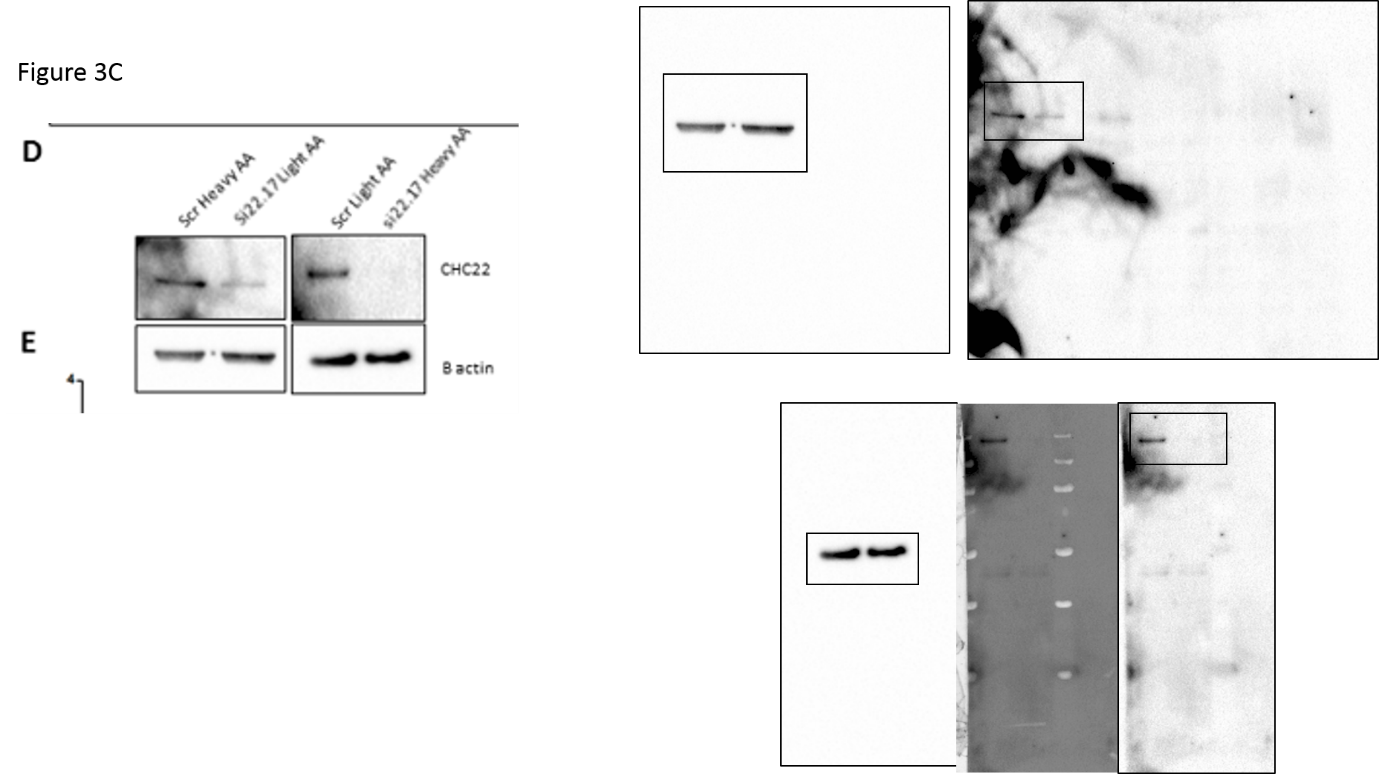


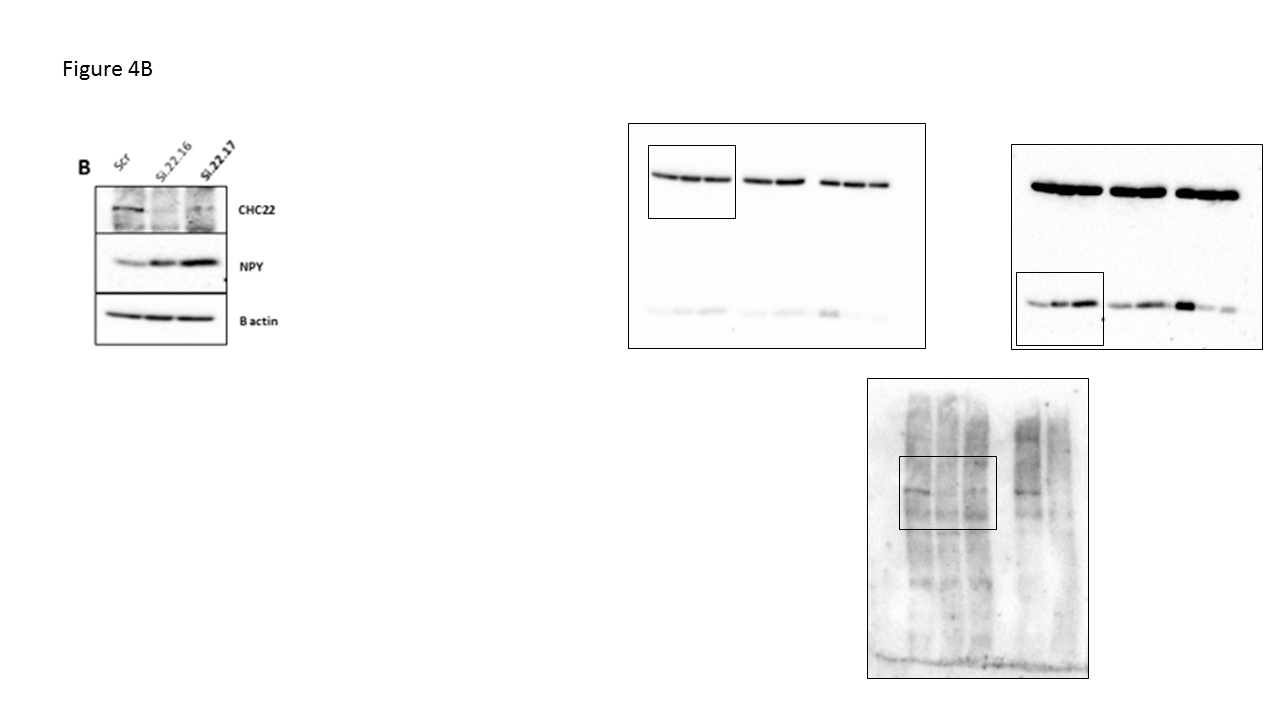


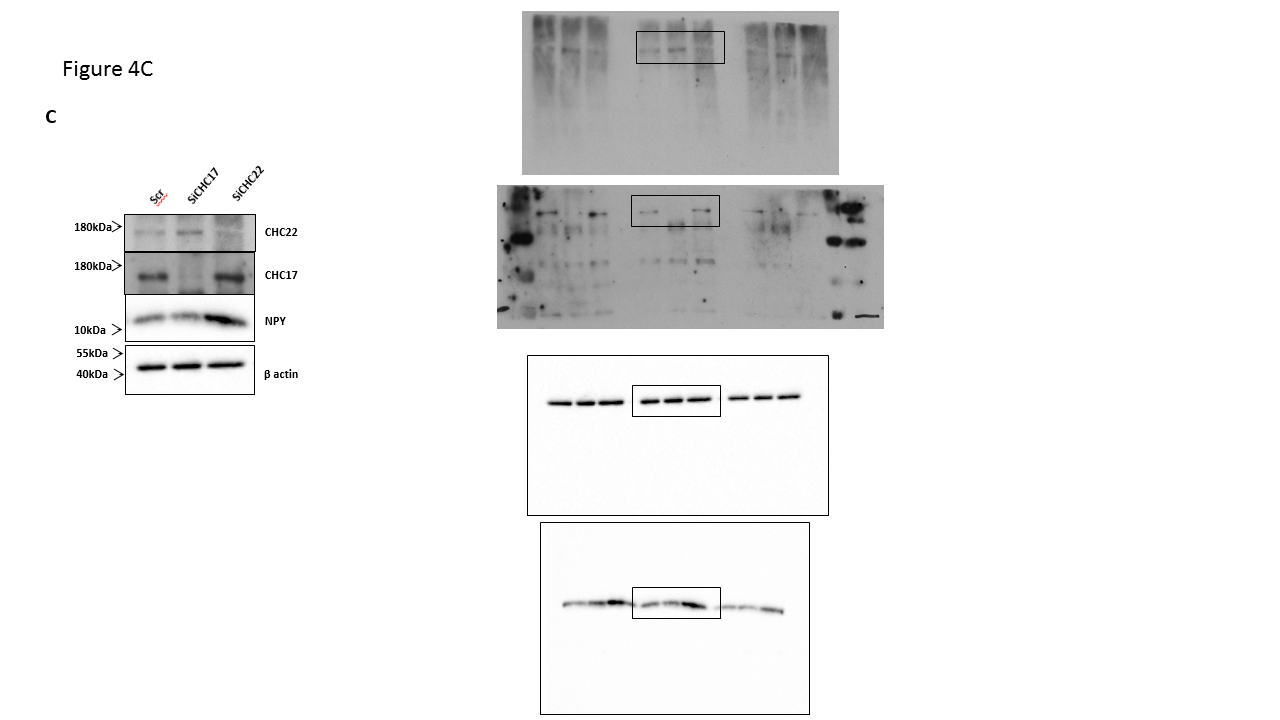


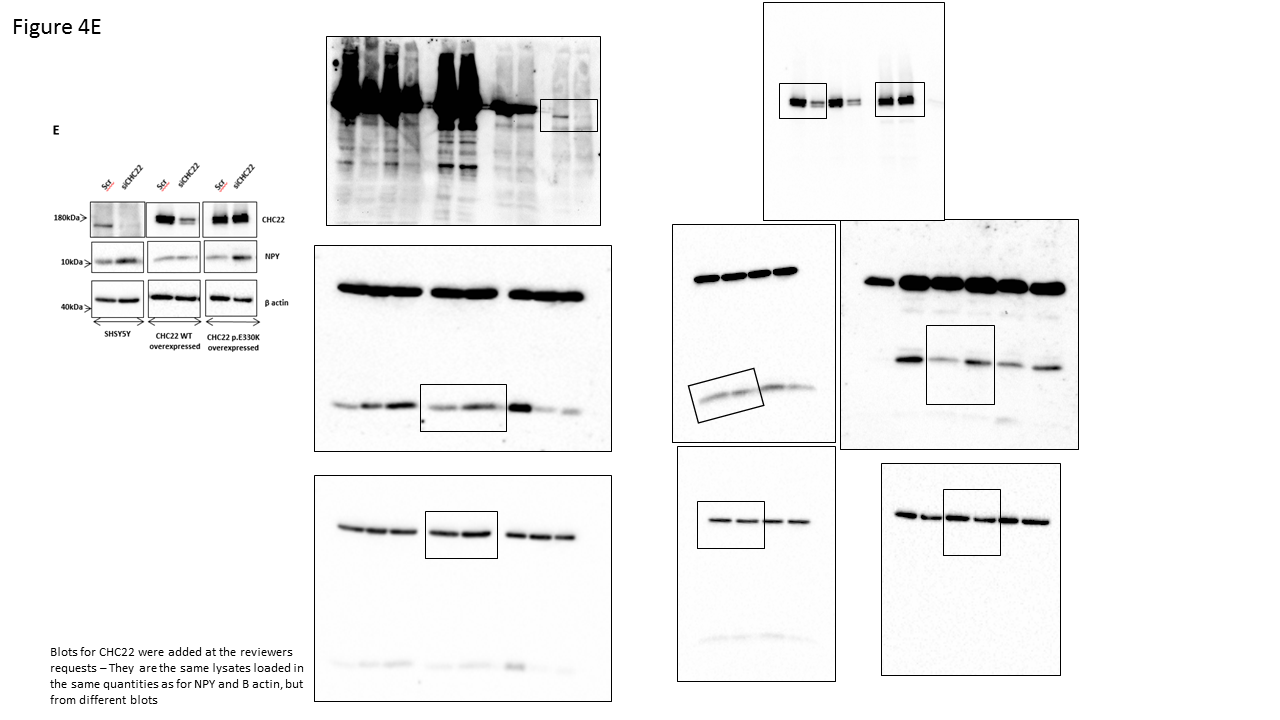


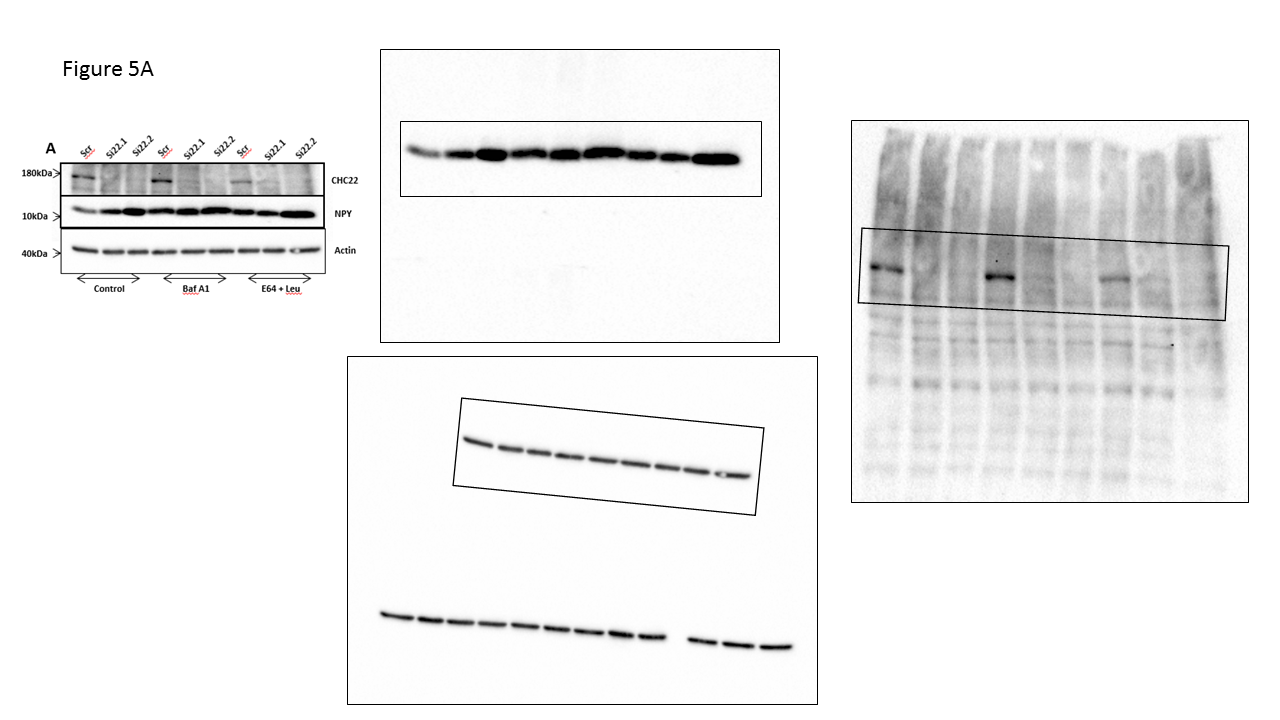


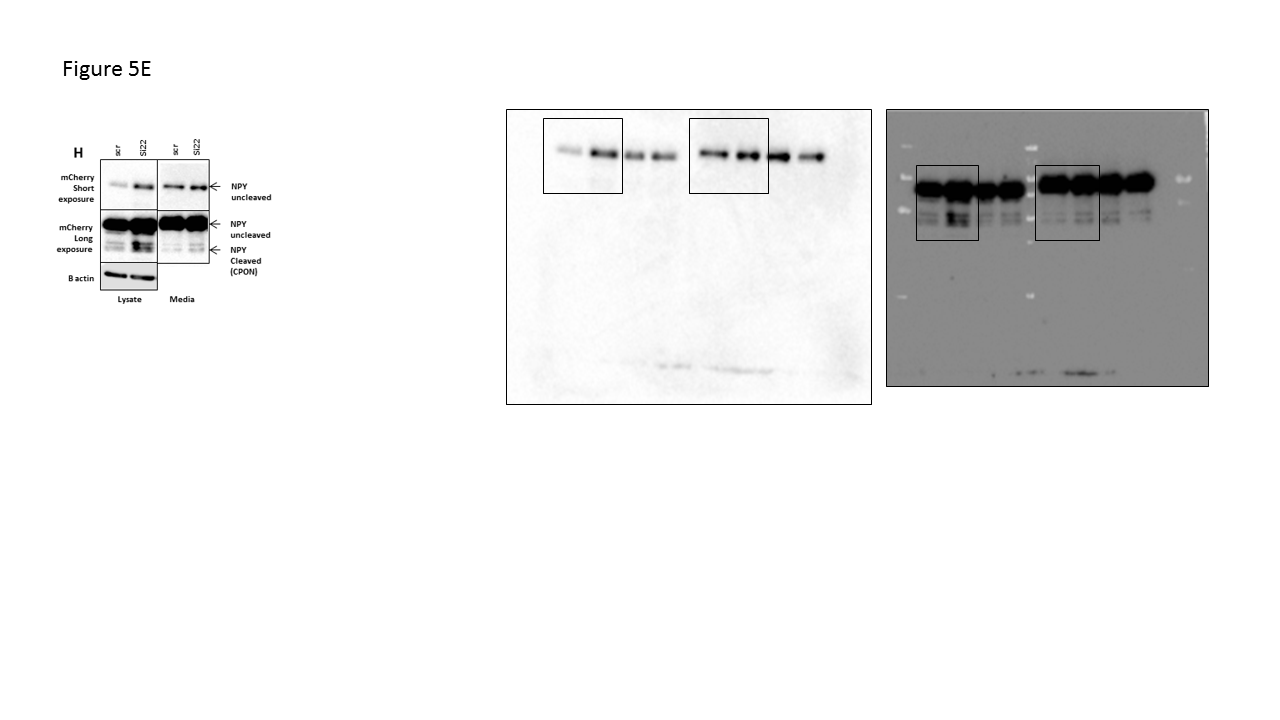


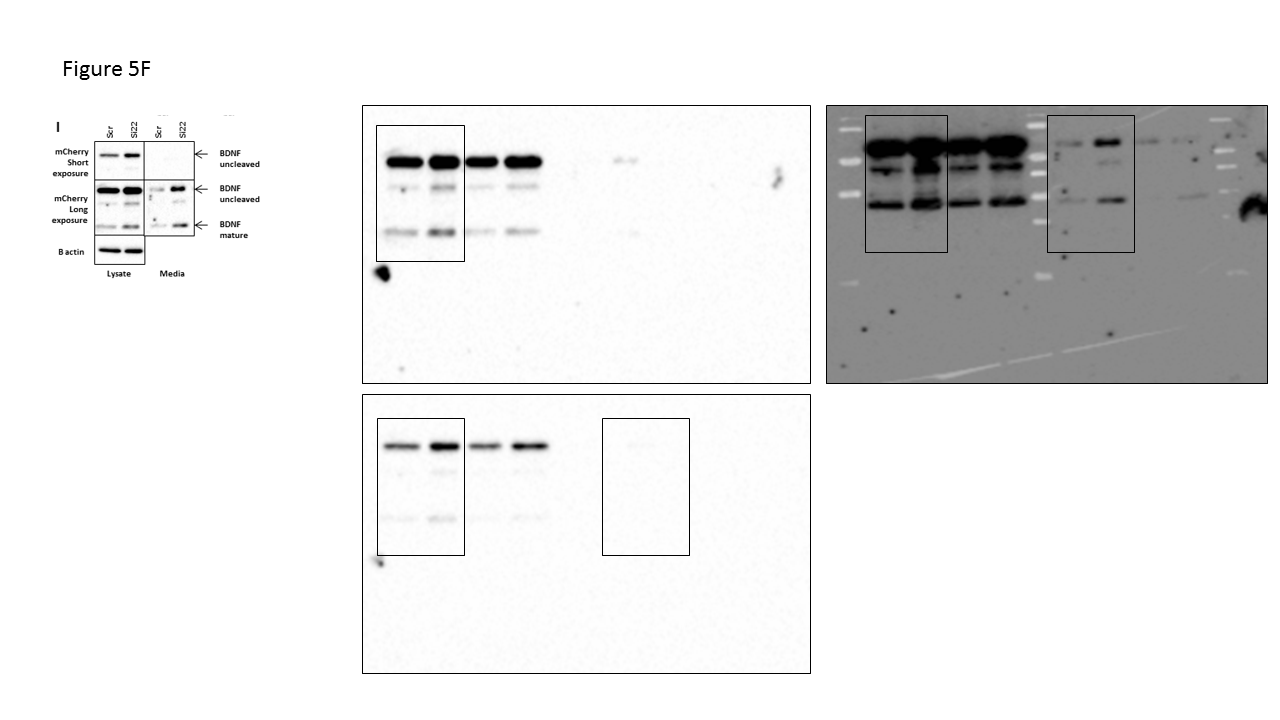

Supplement: Supplementary file 1 — Supplementary Information [file 41598_2018_19980_MOESM1_ESM.doc]
